# Supplementary material for: Climate and water-table levels regulate peat accumulation rates across Europe
Source: PLoS One. 2025 Jul 23;20(7):e0327422. doi: 10.1371/journal.pone.0327422 (PMC12286369; doi:10.1371/journal.pone.0327422)
Supplement: S1 File — Supporting Information 1 (Figure): Bayesian age-depth models. Supporting Information 2 (Table): Spearman’s rank correlation information (Rs (bottom left) and p-values (top right) are shown). Supporting Information 3 (Figure): Boxplot showing aPAR for each region (Britain and Ireland, Continental Europe; Scandinavia and Baltics).Supporting Information 4 (Figure): Boxplot showing aPAR across different climate phases including the Little Ice Age (LIA: 1500–1850 CE), Medieval Warm Period (MWP: 950–1250 CE) and Roman Warm Period (RWP: 1–400 CE). All data points are shows as well as the non-Little Ice Age data points (nLIA). Supporting Information 5 (Figure): Theil–Sen robust regression scatterplot of aPAR versus modern climatic data (NOAA–CIRES–DOE 20th Century Reanalysis Version 3). Supporting Information 6 (Figure): Theil–Sen robust regression scatterplot of aPAR versus palaeo-climatic data (CHELSA-TraCE21k). Supporting Information 7 (Table): Site-based data for aPAR, WTD, contemporary climate and palaeo-climate. Supporting Information 8 (Table): Theil–Sen robust regression information for aPAR regressions with WTD, contemporary climate, and palaeo-climate. Supporting Information 9 (Dataset). All data. (ZIP) [file pone.0327422.s001.zip › SI_1_ADmods.pdf]

Supplementary information

Bayesian age-depth models

Guide to age-depth model figures:

Upper panels depict the MCMC iterations (left; good runs show a stationary distribution with little structure among neighbouring iterations), the prior (green curves) and posterior (grey histograms) distributions for the accumulation rate (middle panel) and memory (right panel). Bottom panel shows the calibrated ^{14}C dates (transparent blue) and the age-depth model (darker greys indicate more likely calendar ages; grey stippled lines show 95% confidence intervals; red curve shows single 'best' model based on the mean age for each depth).

Prior information for accumulation rate and its memory or variability are shown as red text.

Please refer to http://www.chrono.qub.ac.uk/blaauw/manualBacon_2.3.pdf
and Blaauw and Christen (2011) for further information.

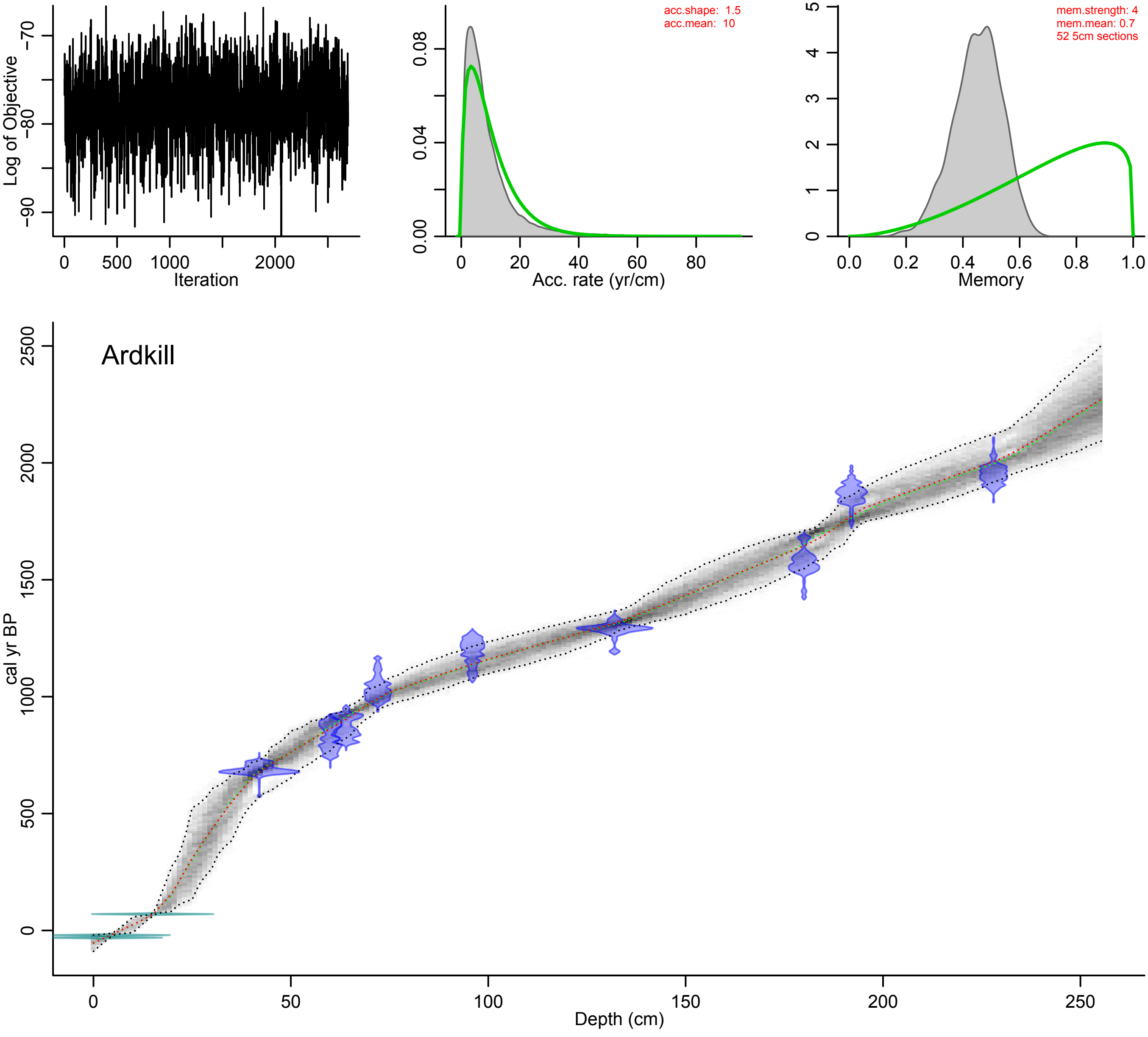

Log of Objective
Iteration
acc.shape: 1.5
acc.mean: 10
Acc. rate (yr/cm)
mem.strength: 4
mem.mean: 0.7
52 5cm sections
Memory
Ardkill
cal yr BP
Depth (cm)

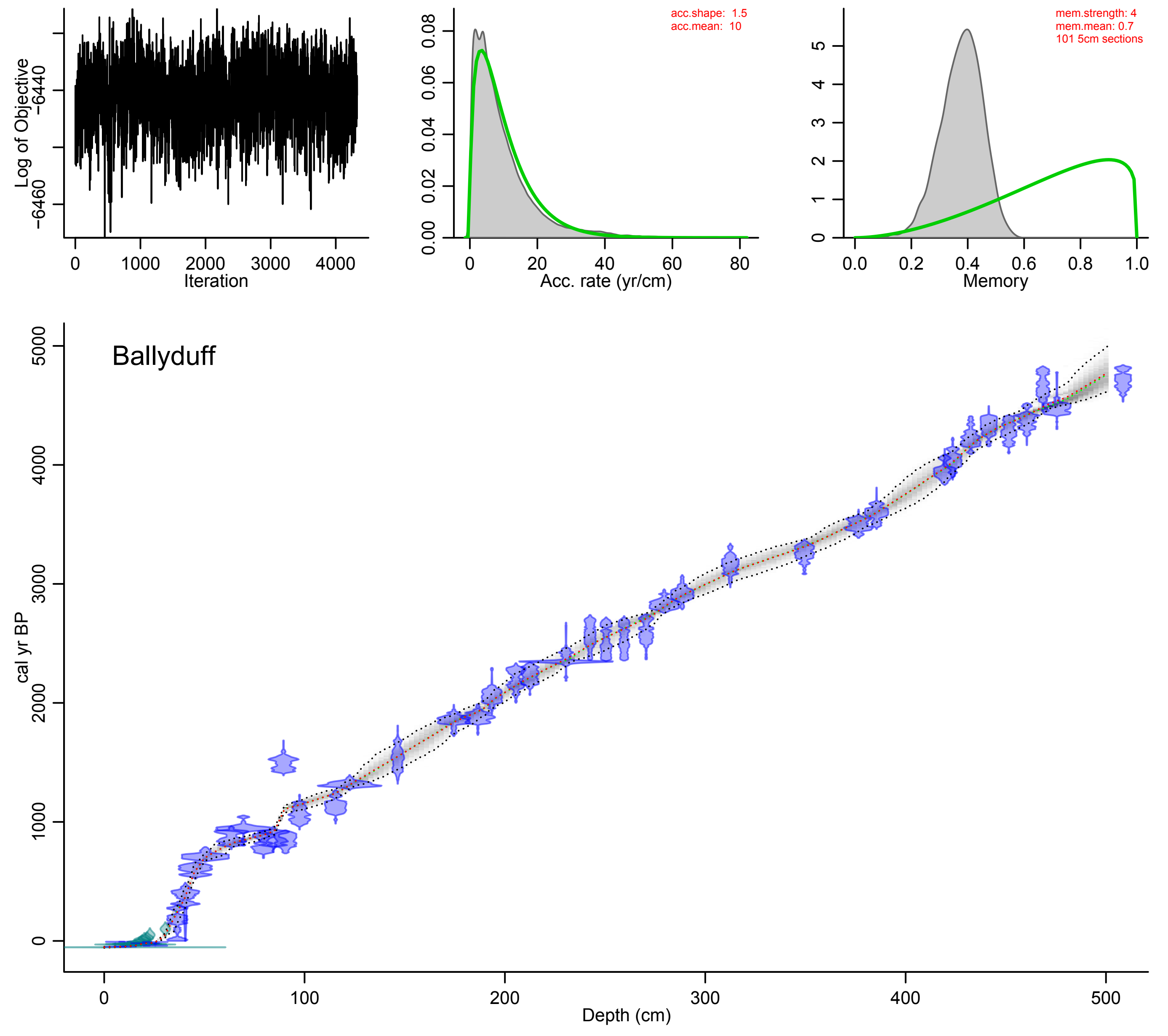
Log of Objective
−6440
−6460
0
1000
2000
3000
4000
Iteration
acc.shape: 1.5
acc.mean: 10
0.00
0.02
0.04
0.06
0.08
0
20
40
60
80
Acc. rate (yr/cm)
mem.strength: 4
mem.mean: 0.7
101 5cm sections
0
1
2
3
4
5
0.0
0.2
0.4
0.6
0.8
1.0
Memory
Ballyduff
cal yr BP
0
1000
2000
3000
4000
5000
0
100
200
300
400
500
Depth (cm)

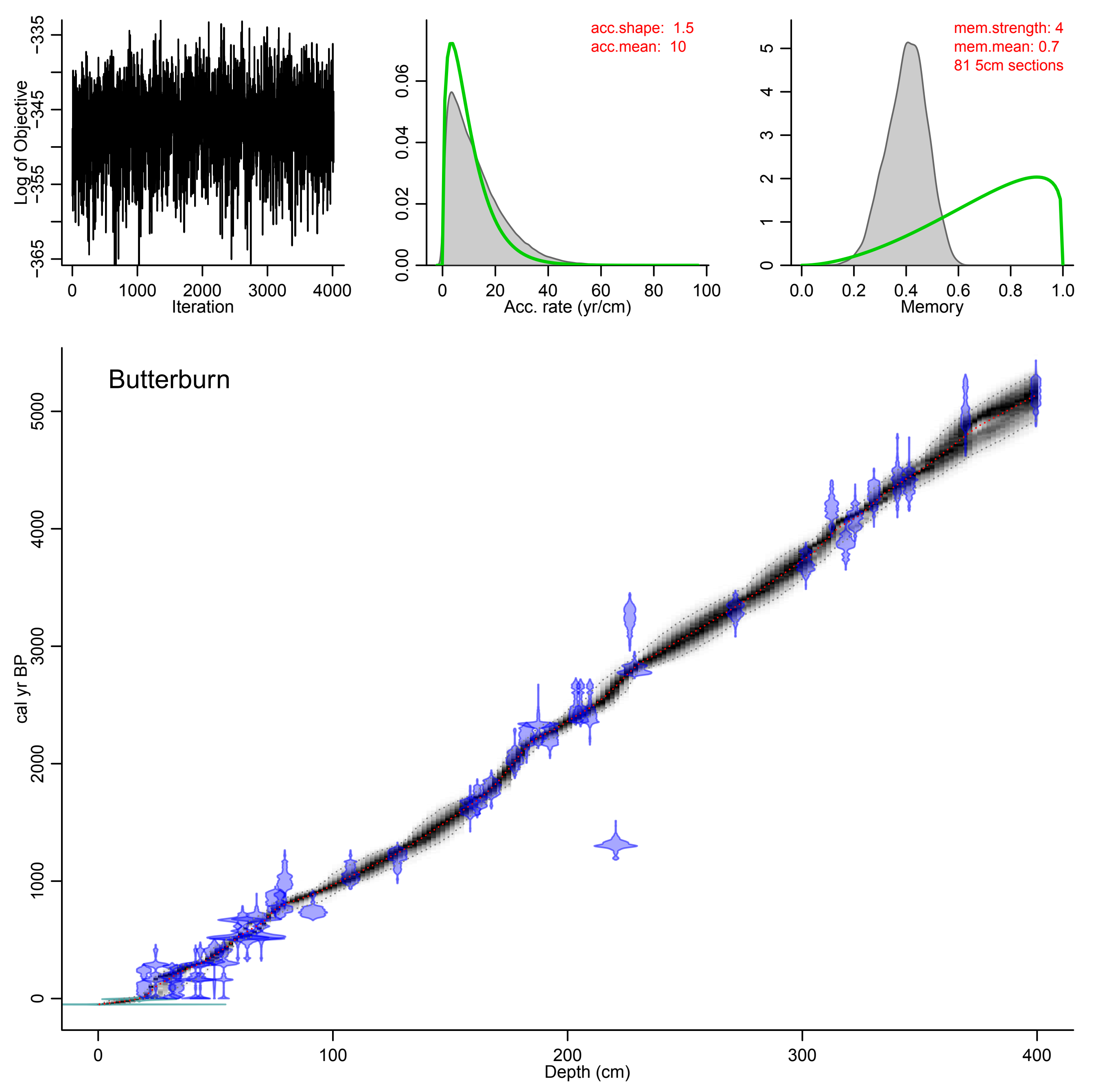

Log of Objective
Iteration
acc.shape: 1.5
acc.mean: 10
Acc. rate (yr/cm)
mem.strength: 4
mem.mean: 0.7
81 5cm sections
Memory
Butterburn
cal yr BP
Depth (cm)

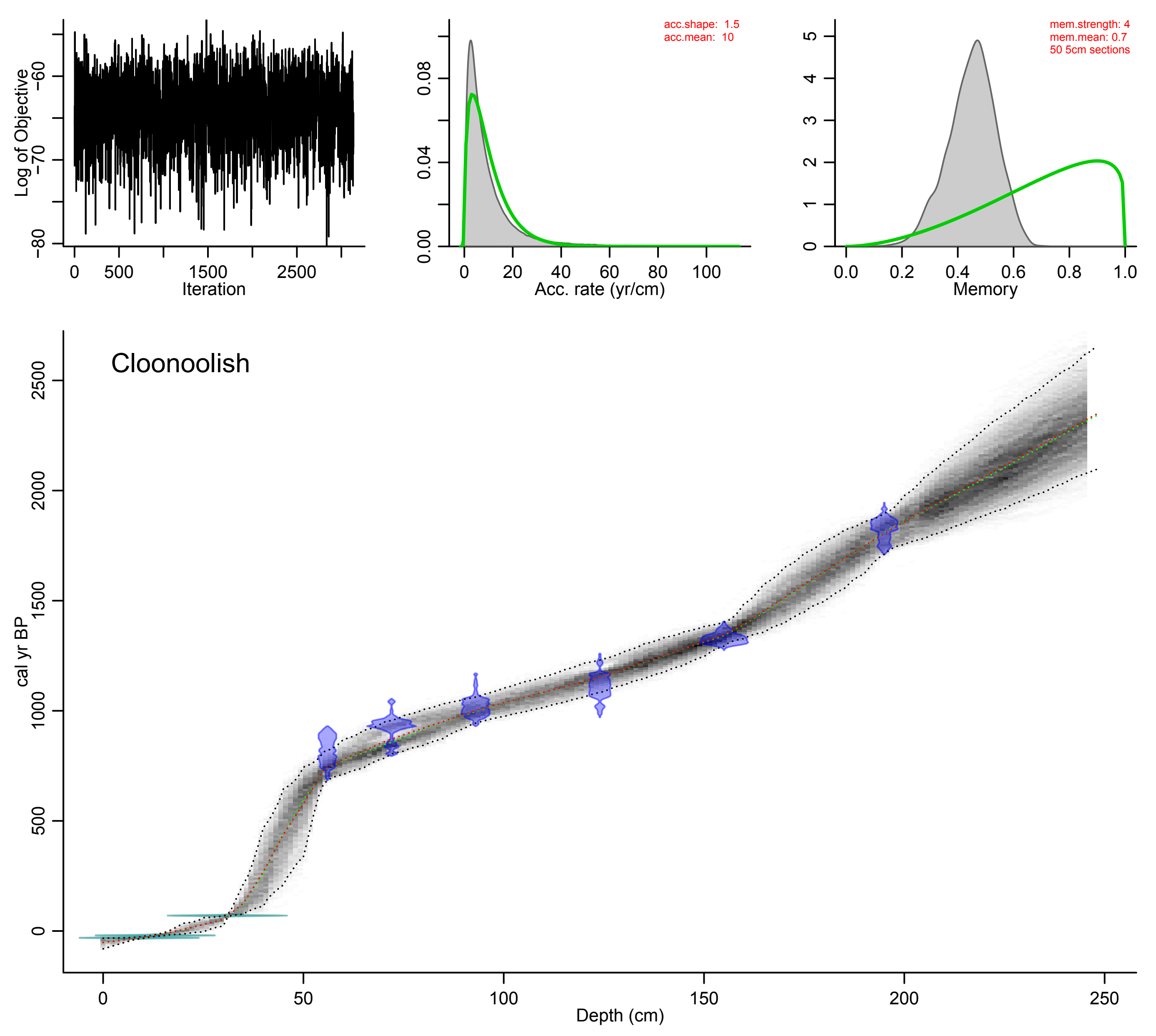

Log of Objective
Iteration
acc.shape: 1.5
acc.mean: 10
Acc. rate (yr/cm)
mem.strength: 4
mem.mean: 0.7
50 5cm sections
Memory
Cloonoolish
cal yr BP
Depth (cm)

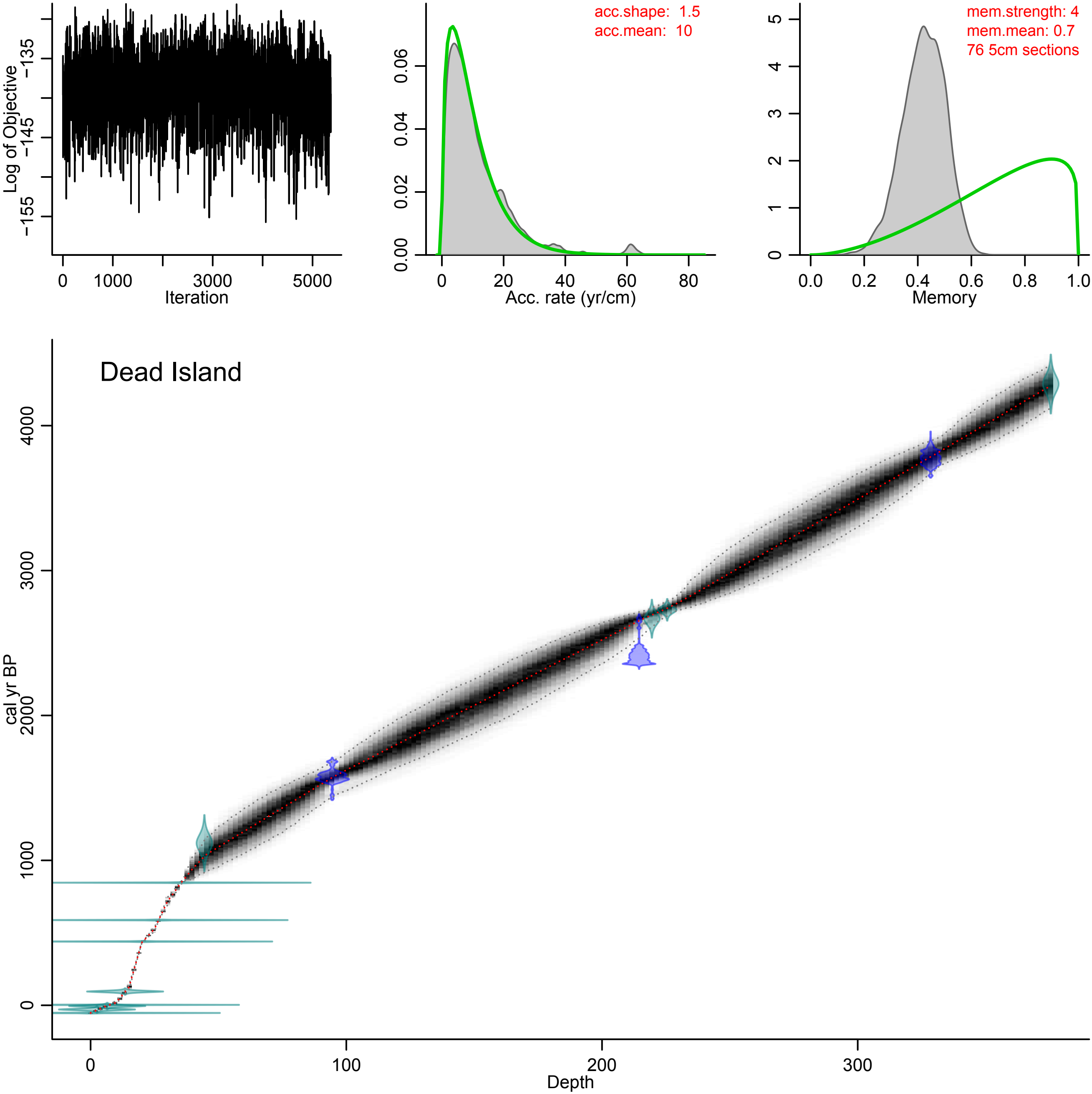

Log of Objective
Iteration
acc.shape: 1.5
acc.mean: 10
Acc. rate (yr/cm)
mem.strength: 4
mem.mean: 0.7
76 5cm sections
Memory
Dead Island
cal yr BP
Depth

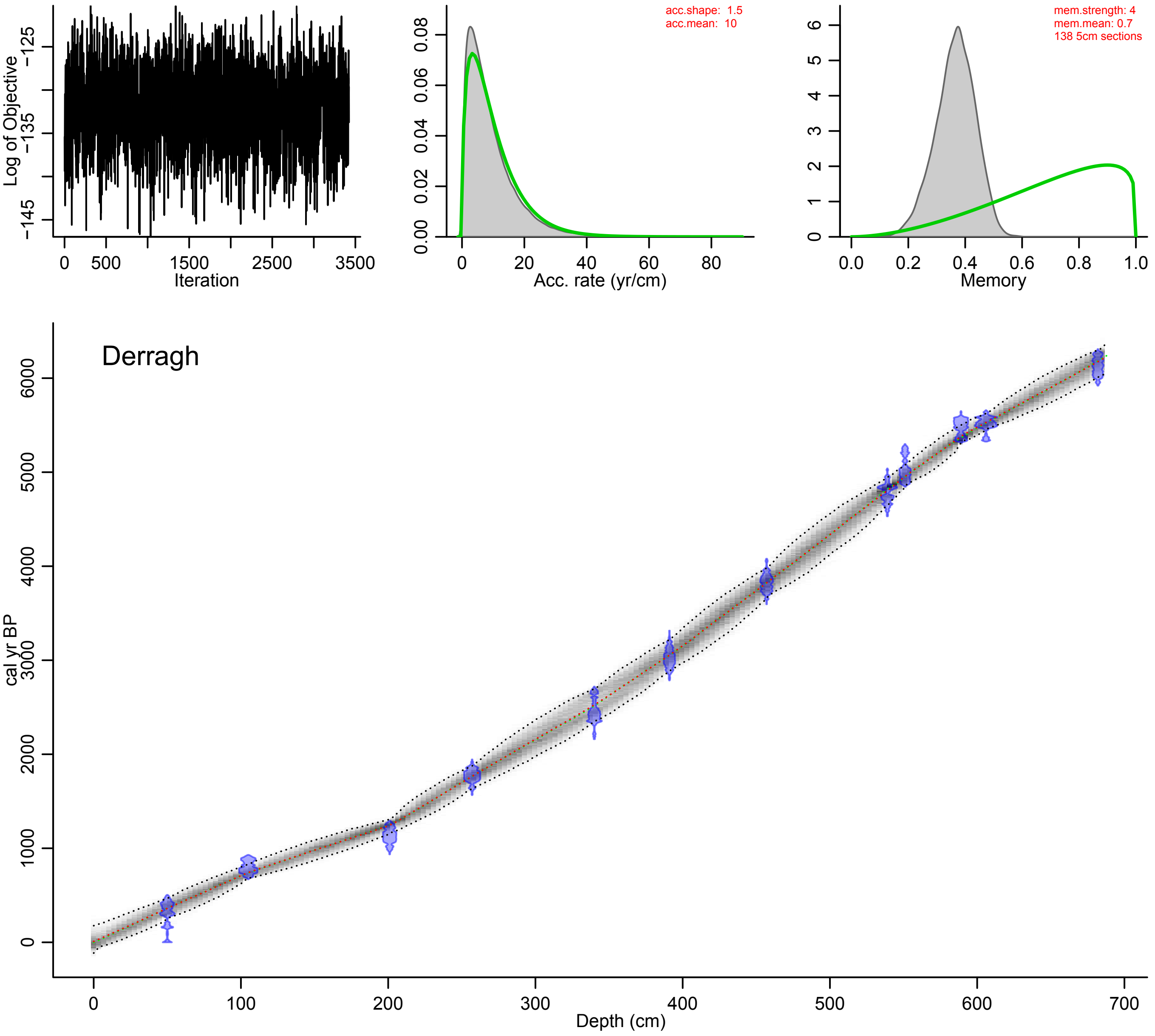

Log of Objective
Iteration
acc.shape: 1.5
acc.mean: 10
Acc. rate (yr/cm)
mem.strength: 4
mem.mean: 0.7
138 5cm sections
Memory
Derragh
cal yr BP
Depth (cm)

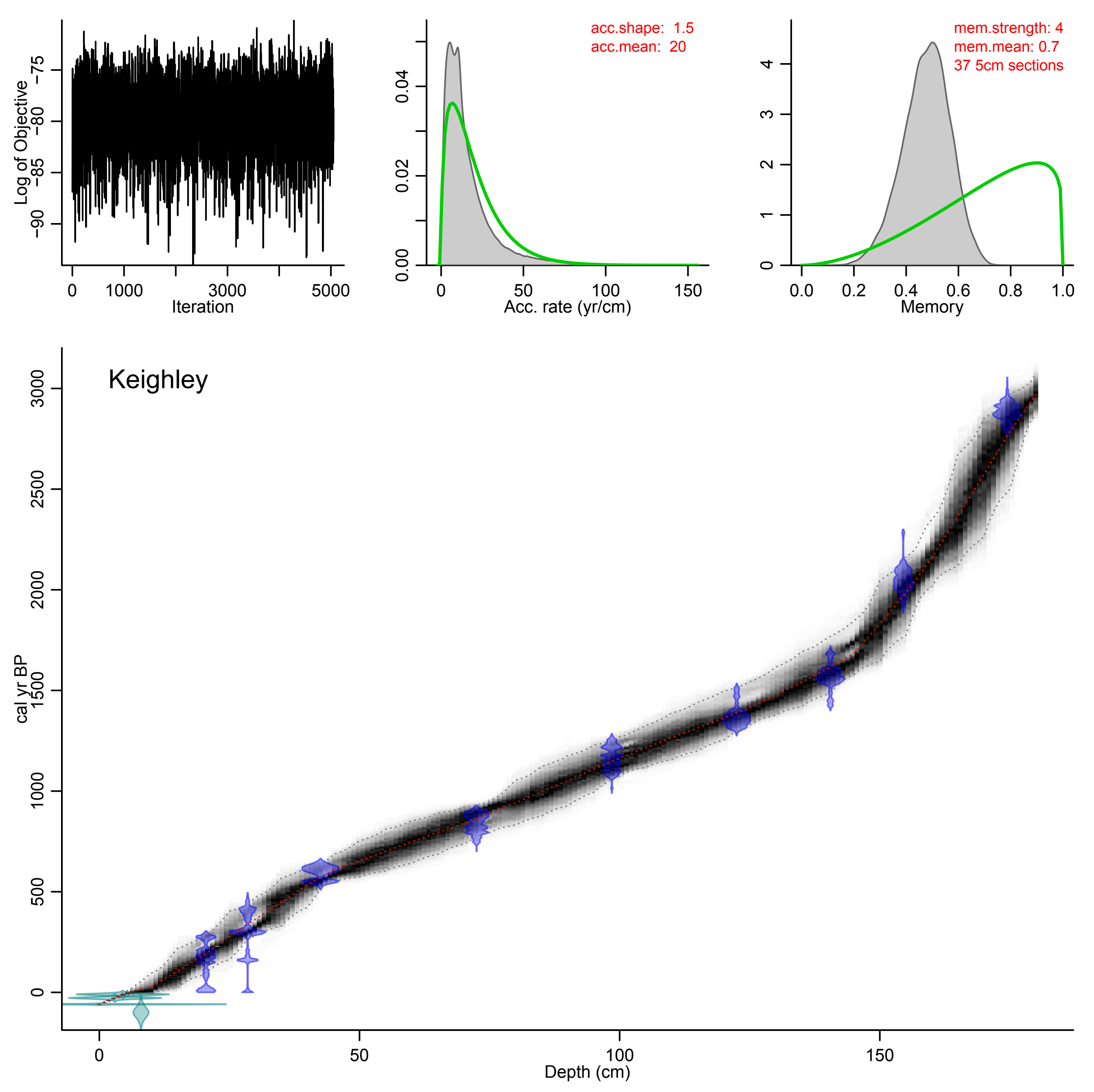

Log of Objective
Iteration
acc.shape: 1.5
acc.mean: 20
Acc. rate (yr/cm)
mem.strength: 4
mem.mean: 0.7
37 5cm sections
Memory
Keighley
cal yr BP
Depth (cm)

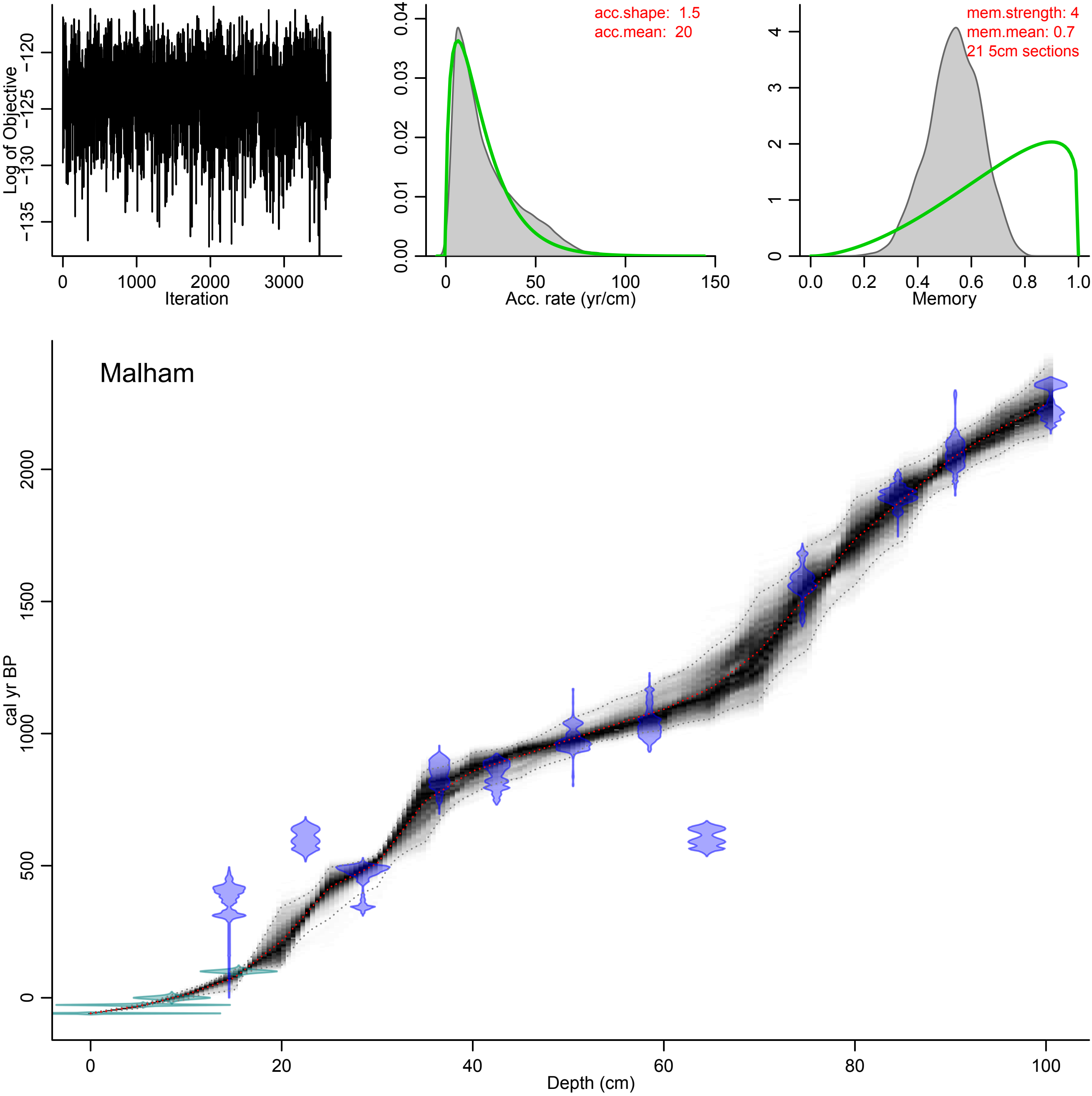

Log of Objective
Iteration
acc.shape: 1.5
acc.mean: 20
Acc. rate (yr/cm)
mem.strength: 4
mem.mean: 0.7
21 5cm sections
Memory
Malham
cal yr BP
Depth (cm)

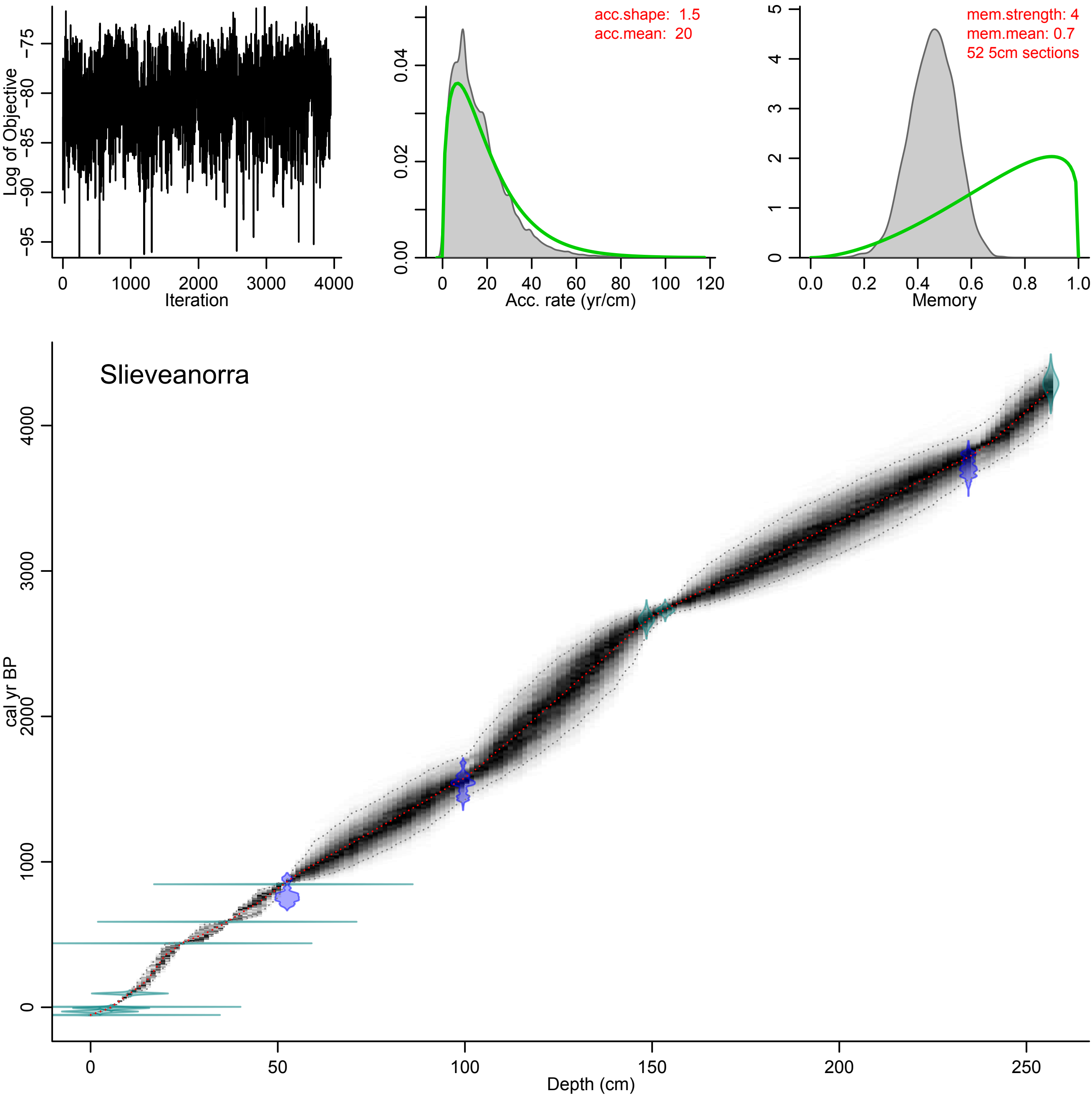

Log of Objective
Iteration
acc.shape: 1.5
acc.mean: 20
Acc. rate (yr/cm)
mem.strength: 4
mem.mean: 0.7
52 5cm sections
Memory
Slieveanorra
cal yr BP
Depth (cm)

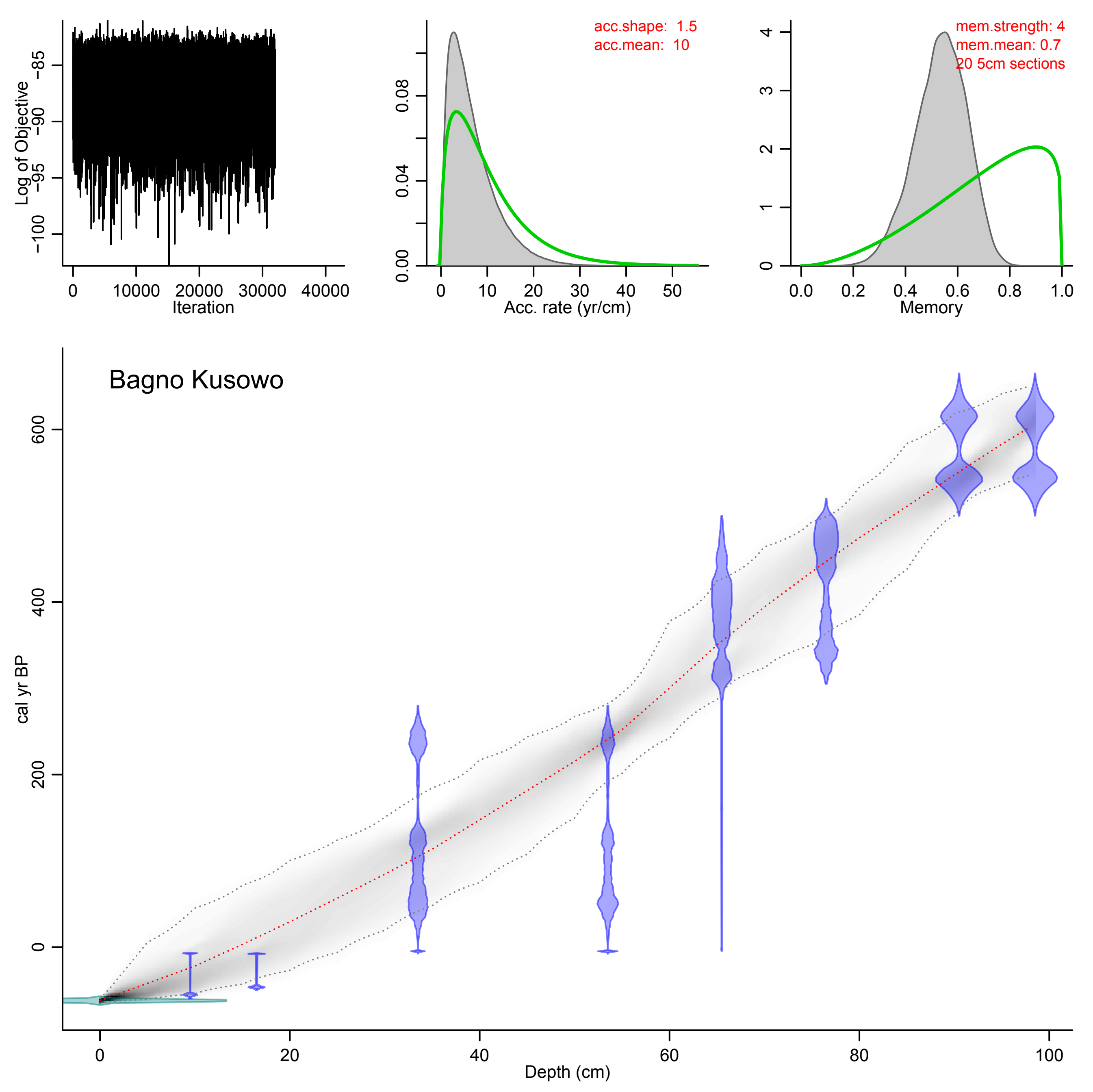

Log of Objective
Iteration
acc.shape: 1.5
acc.mean: 10
Acc. rate (yr/cm)
mem.strength: 4
mem.mean: 0.7
20 5cm sections
Memory
Bagno Kusowo
cal yr BP
Depth (cm)

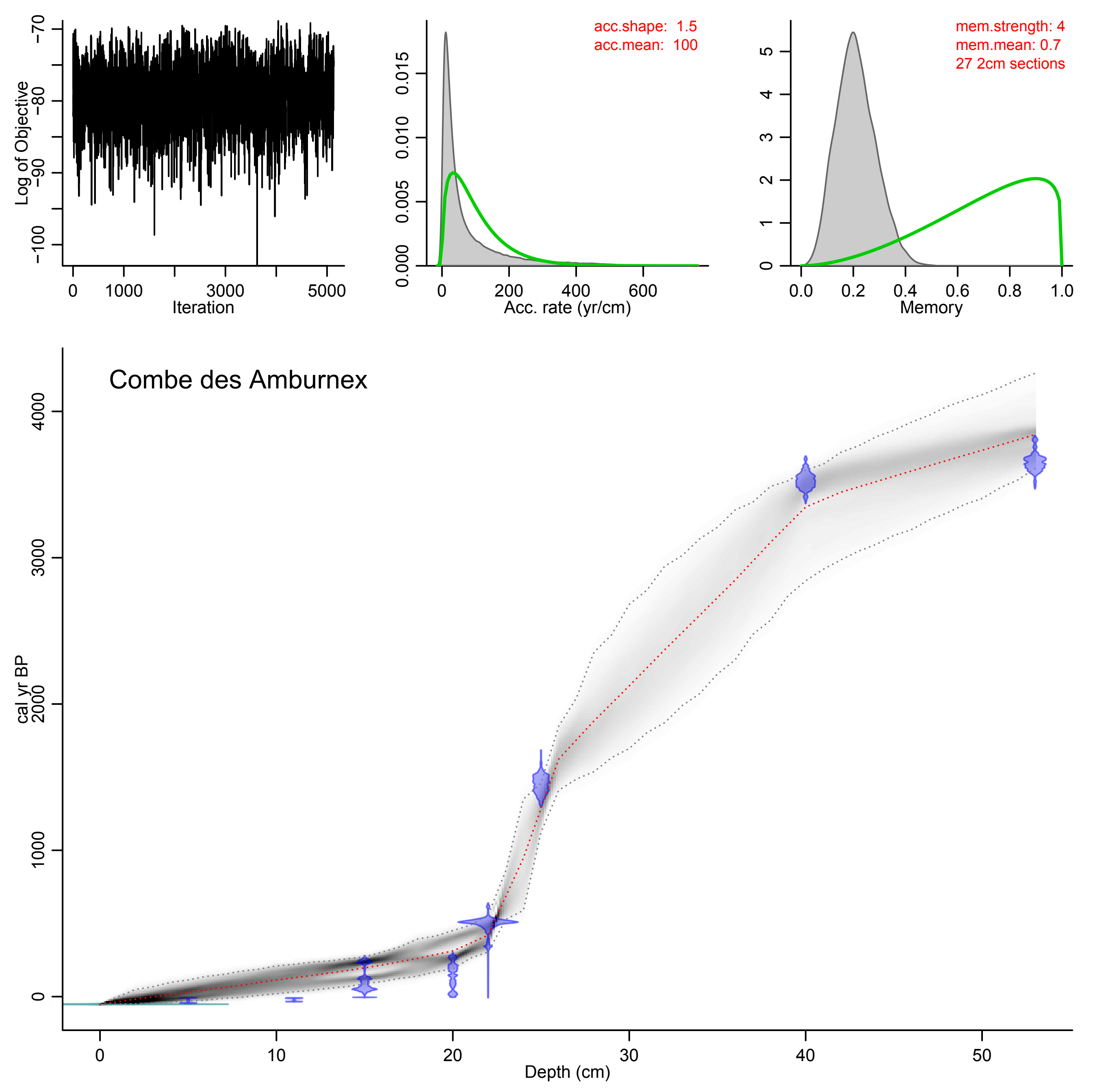
Log of Objective
Iteration
acc.shape: 1.5
acc.mean: 100
Acc. rate (yr/cm)
mem.strength: 4
mem.mean: 0.7
27 2cm sections
Memory
Combe des Amburnex
cal yr BP
Depth (cm)

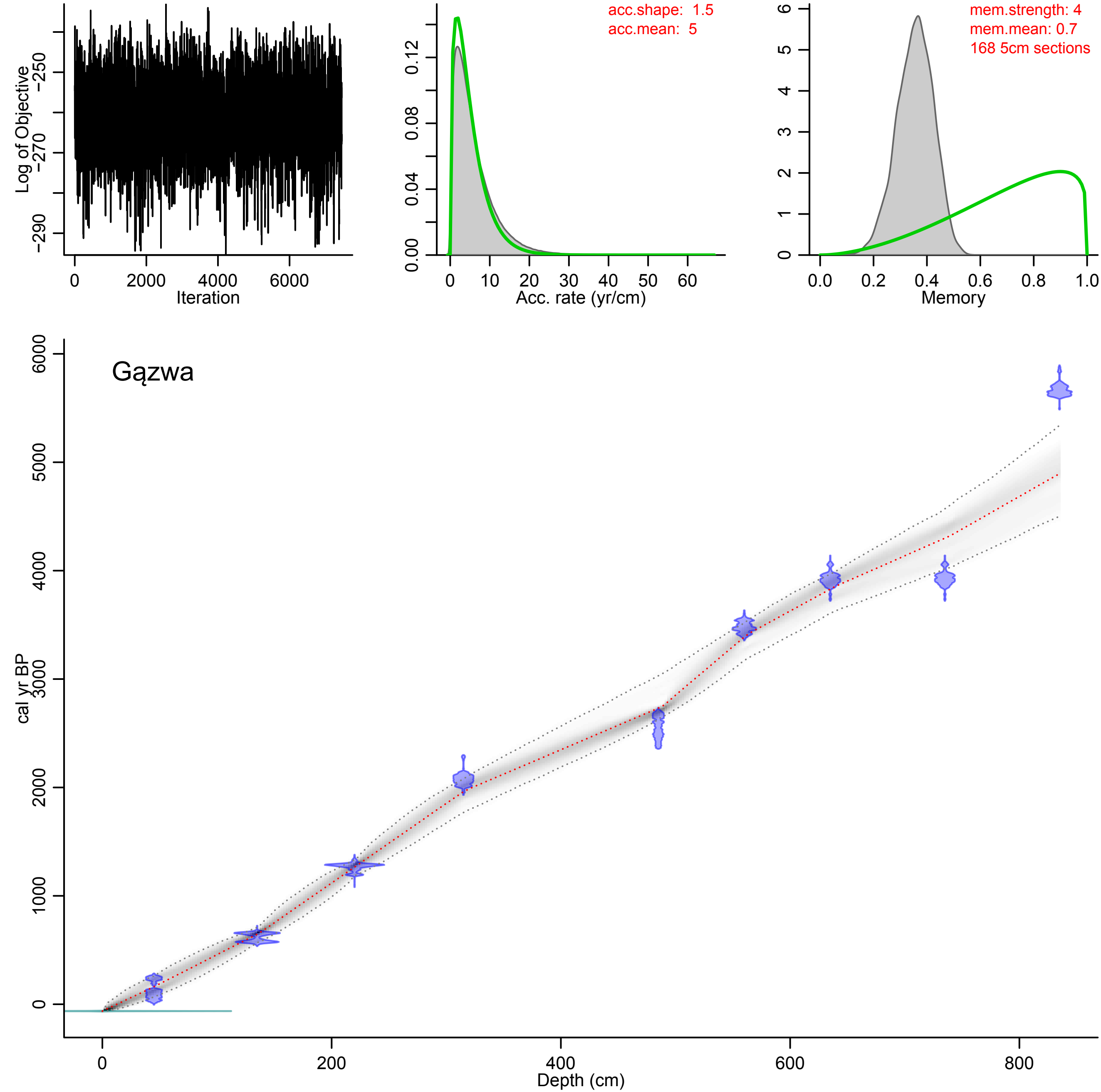

Log of Objective
−250
−270
−290
0
2000
4000
6000
Iteration
acc.shape: 1.5
acc.mean: 5
0.00
0.04
0.08
0.12
0
10
20
30
40
50
60
Acc. rate (yr/cm)
mem.strength: 4
mem.mean: 0.7
168 5cm sections
0
1
2
3
4
5
6
0.0
0.2
0.4
0.6
0.8
1.0
Memory
Gązwa
cal yr BP
0
1000
2000
3000
4000
5000
6000
0
200
400
600
800
Depth (cm)

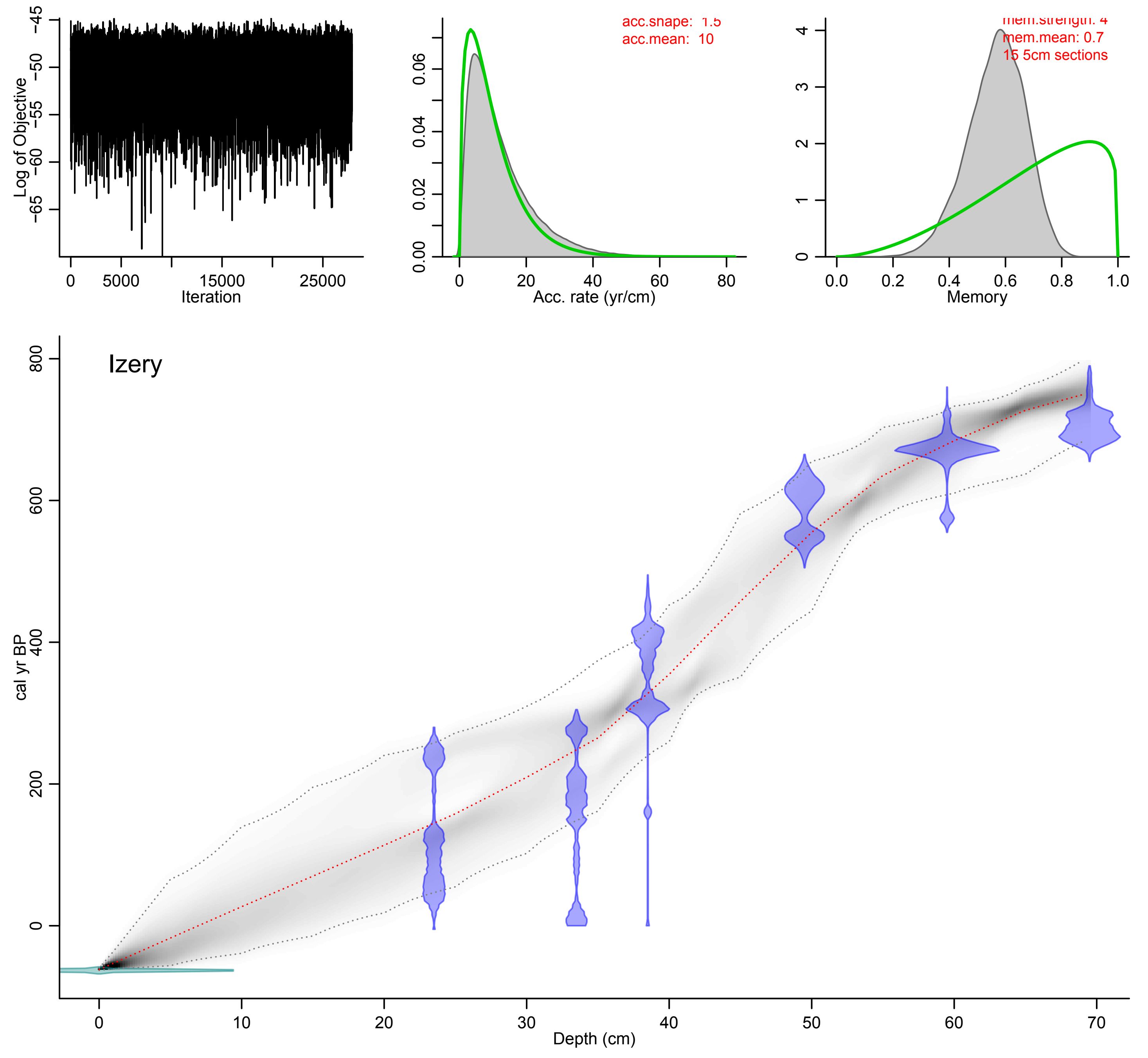

Log of Objective
Iteration
acc.shape: 1.5
acc.mean: 10
Acc. rate (yr/cm)
mem.strength: 4
mem.mean: 0.7
15 5cm sections
Memory
Izery
cal yr BP
Depth (cm)

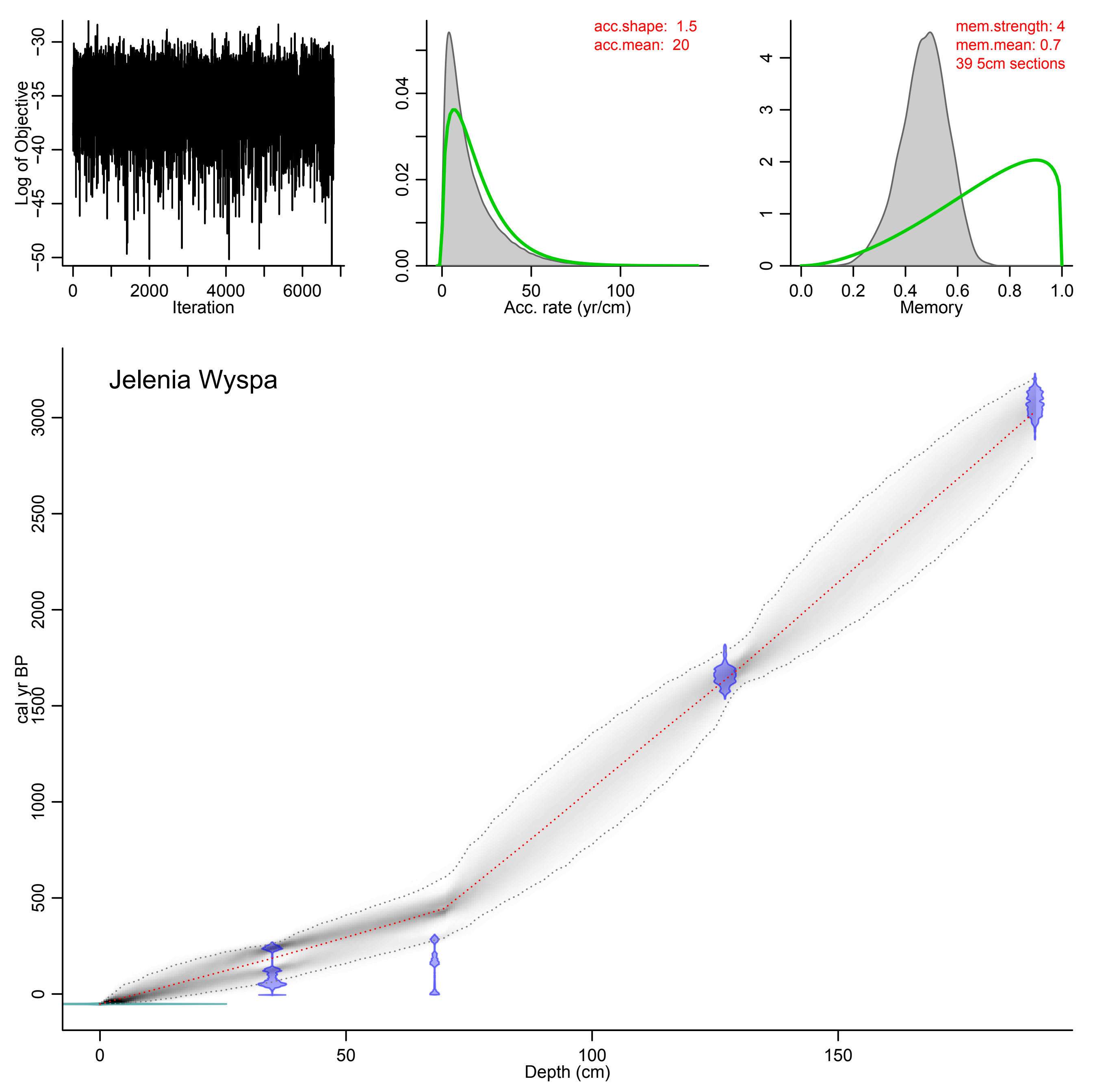

Log of Objective
Iteration
acc.shape: 1.5
acc.mean: 20
Acc. rate (yr/cm)
mem.strength: 4
mem.mean: 0.7
39 5cm sections
Memory
Jelenia Wyspa
cal yr BP
Depth (cm)

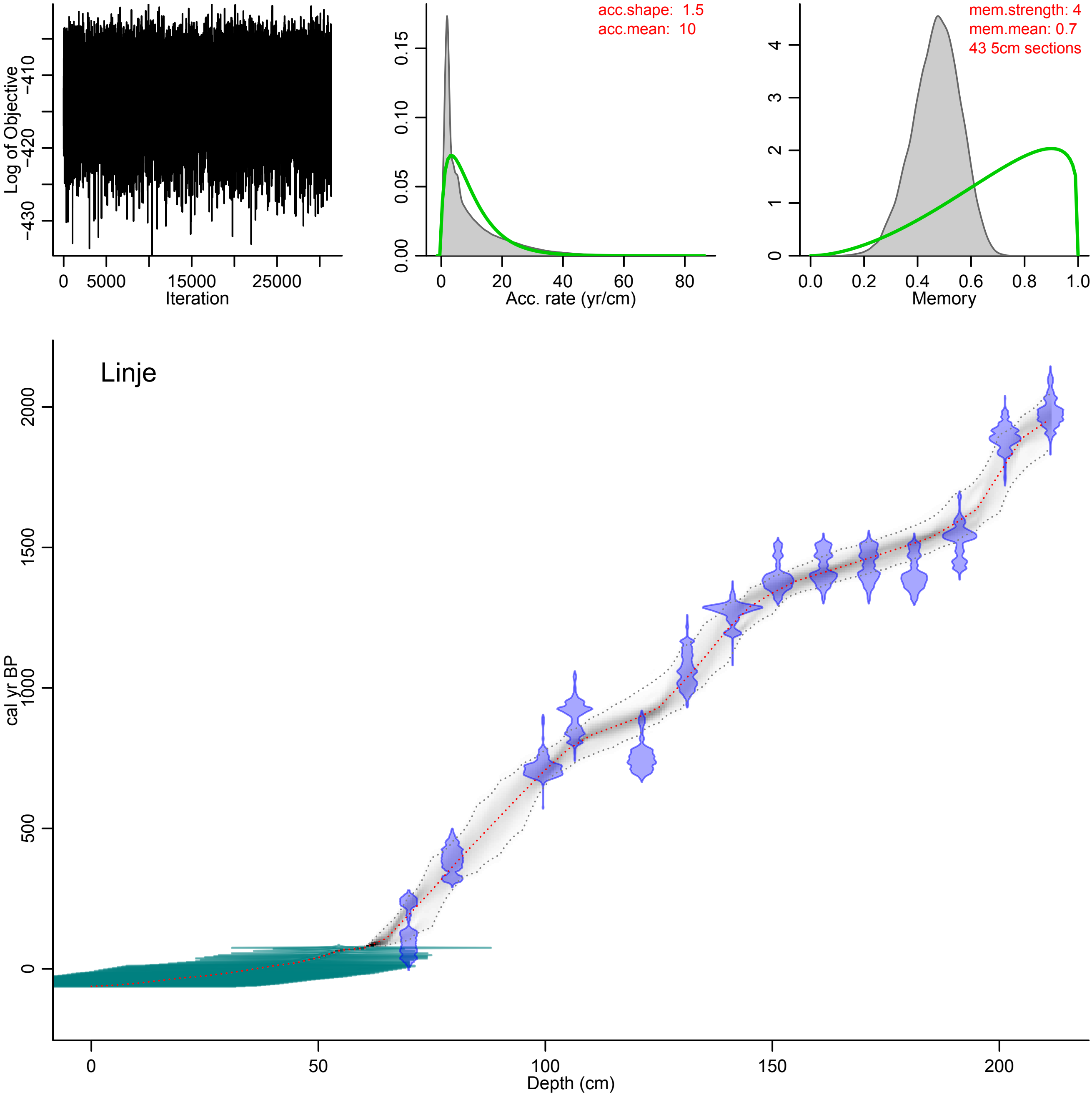

Log of Objective
Iteration
acc.shape: 1.5
acc.mean: 10
Acc. rate (yr/cm)
mem.strength: 4
mem.mean: 0.7
43 5cm sections
Memory
Linje
cal yr BP
Depth (cm)

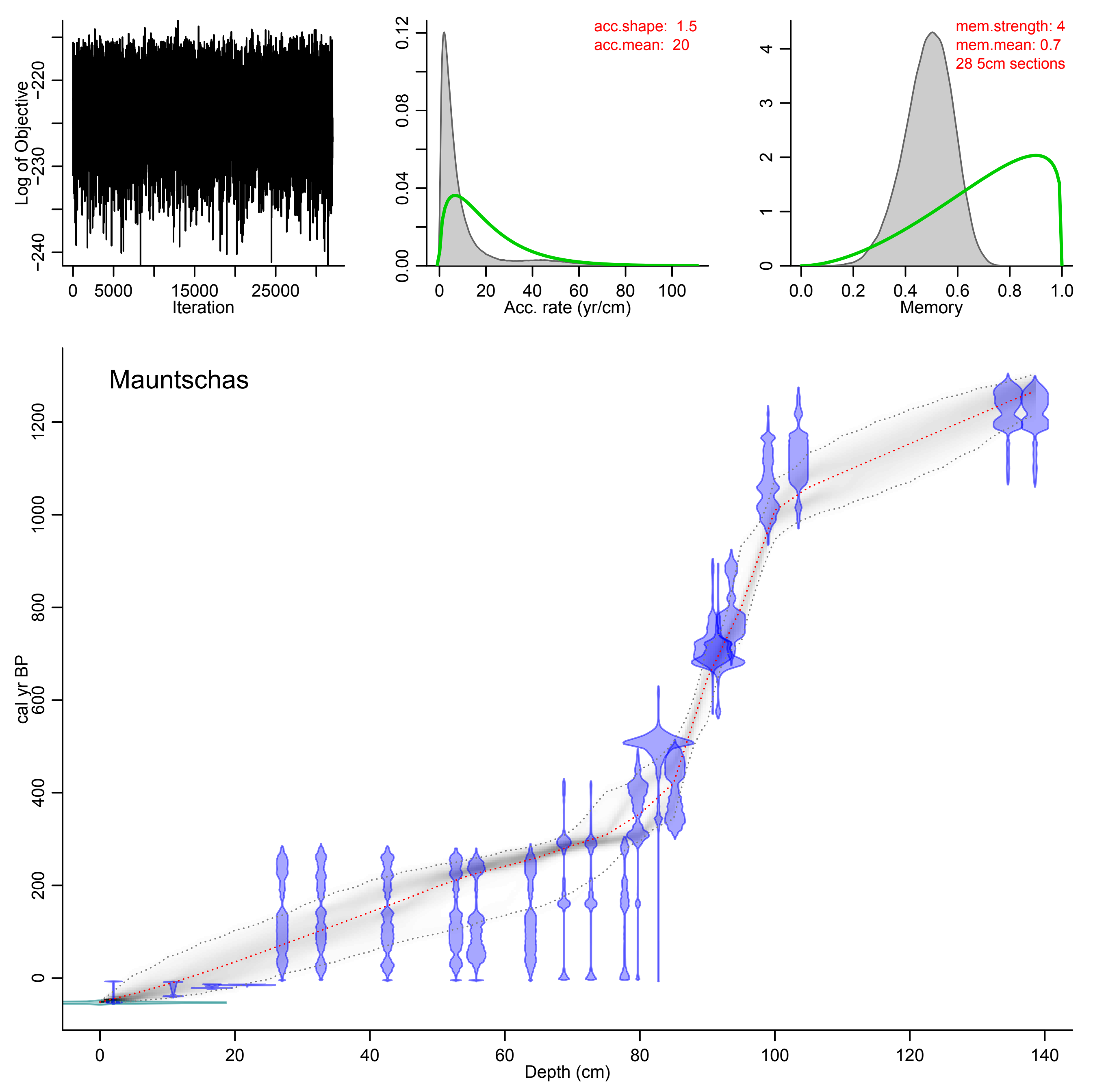

Log of Objective
Iteration
acc.shape: 1.5
acc.mean: 20
Acc. rate (yr/cm)
mem.strength: 4
mem.mean: 0.7
28 5cm sections
Memory
Mauntschas
cal yr BP
Depth (cm)

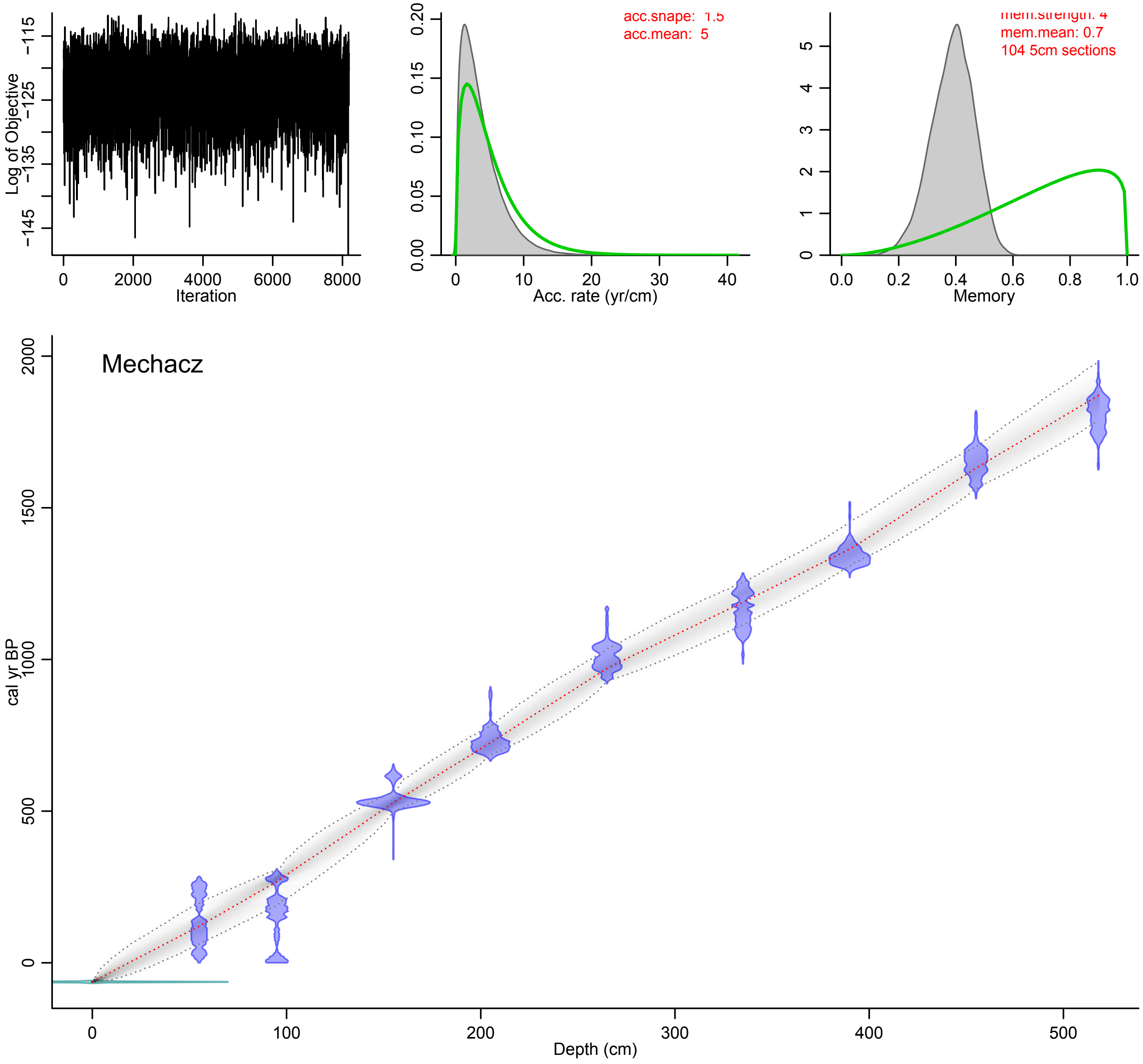

Log of Objective
Iteration
acc.shape: 1.5
acc.mean: 5
Acc. rate (yr/cm)
mem.strength: 4
mem.mean: 0.7
104 5cm sections
Memory
Mechacz
cal yr BP
Depth (cm)

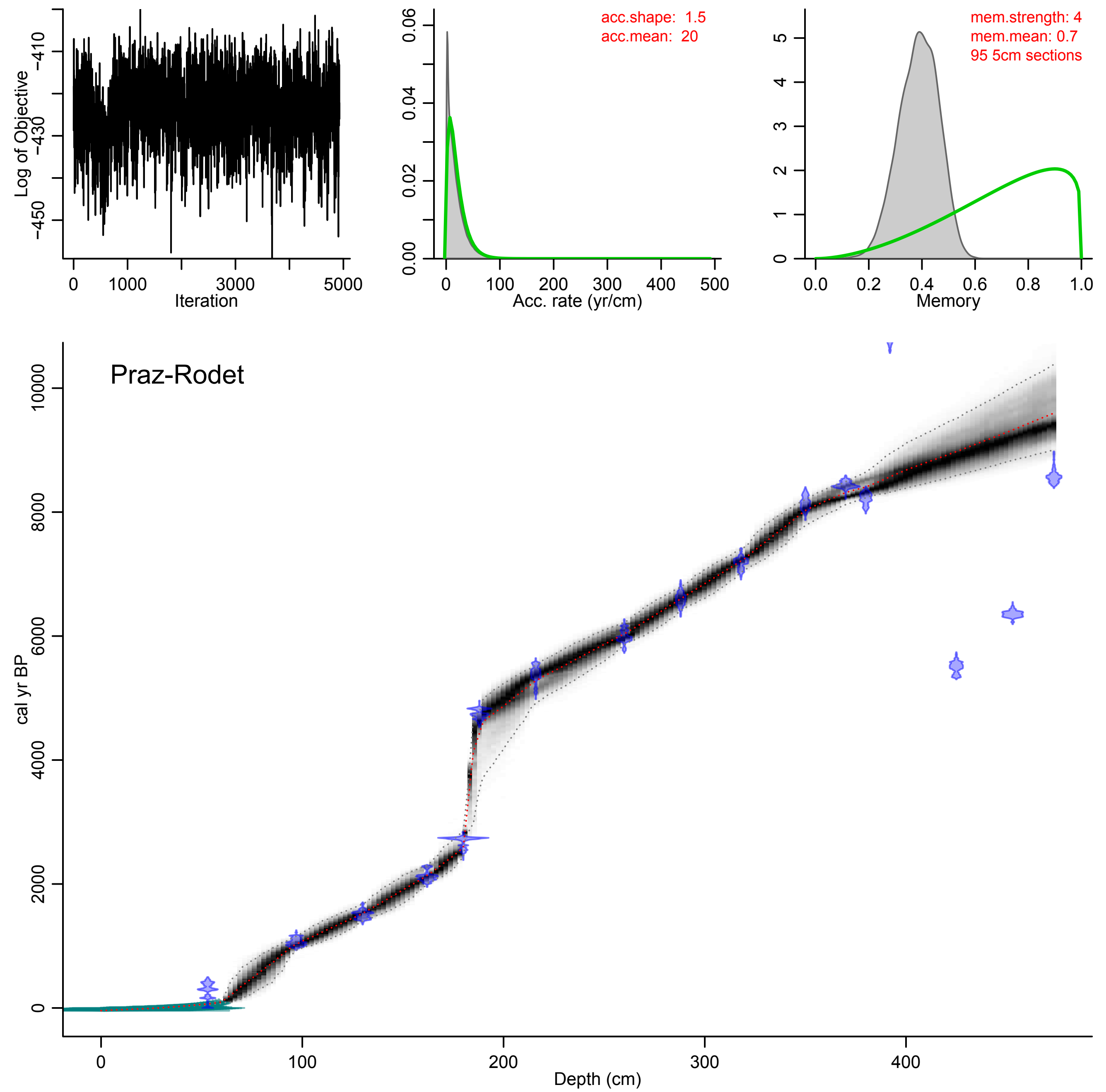

Log of Objective
Iteration
acc.shape: 1.5
acc.mean: 20
Acc. rate (yr/cm)
mem.strength: 4
mem.mean: 0.7
95 5cm sections
Memory
Praz-Rodet
cal yr BP
Depth (cm)

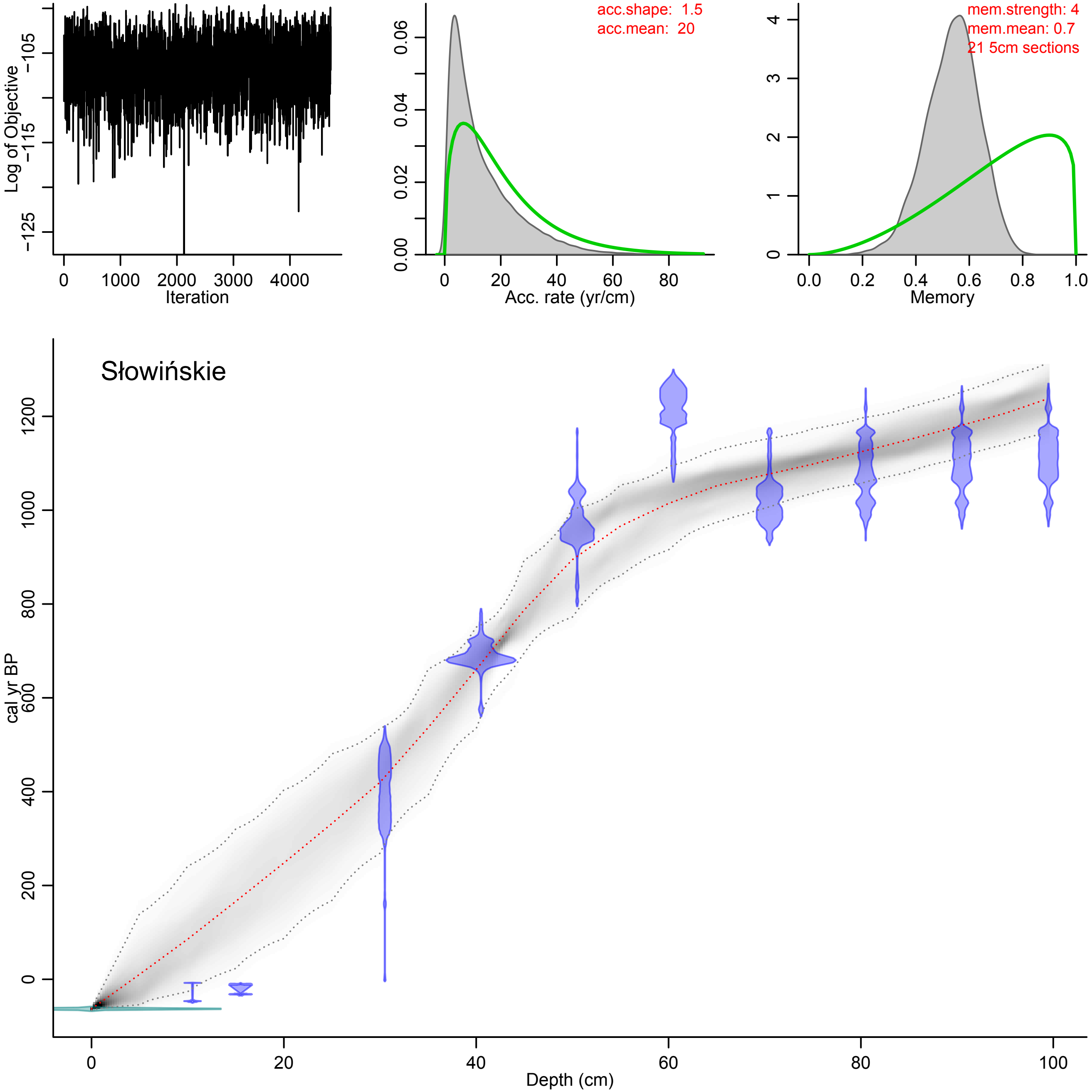
Log of Objective
Iteration
acc.shape: 1.5
acc.mean: 20
Acc. rate (yr/cm)
mem.strength: 4
mem.mean: 0.7
21 5cm sections
Memory
Słowińskie
cal yr BP
Depth (cm)

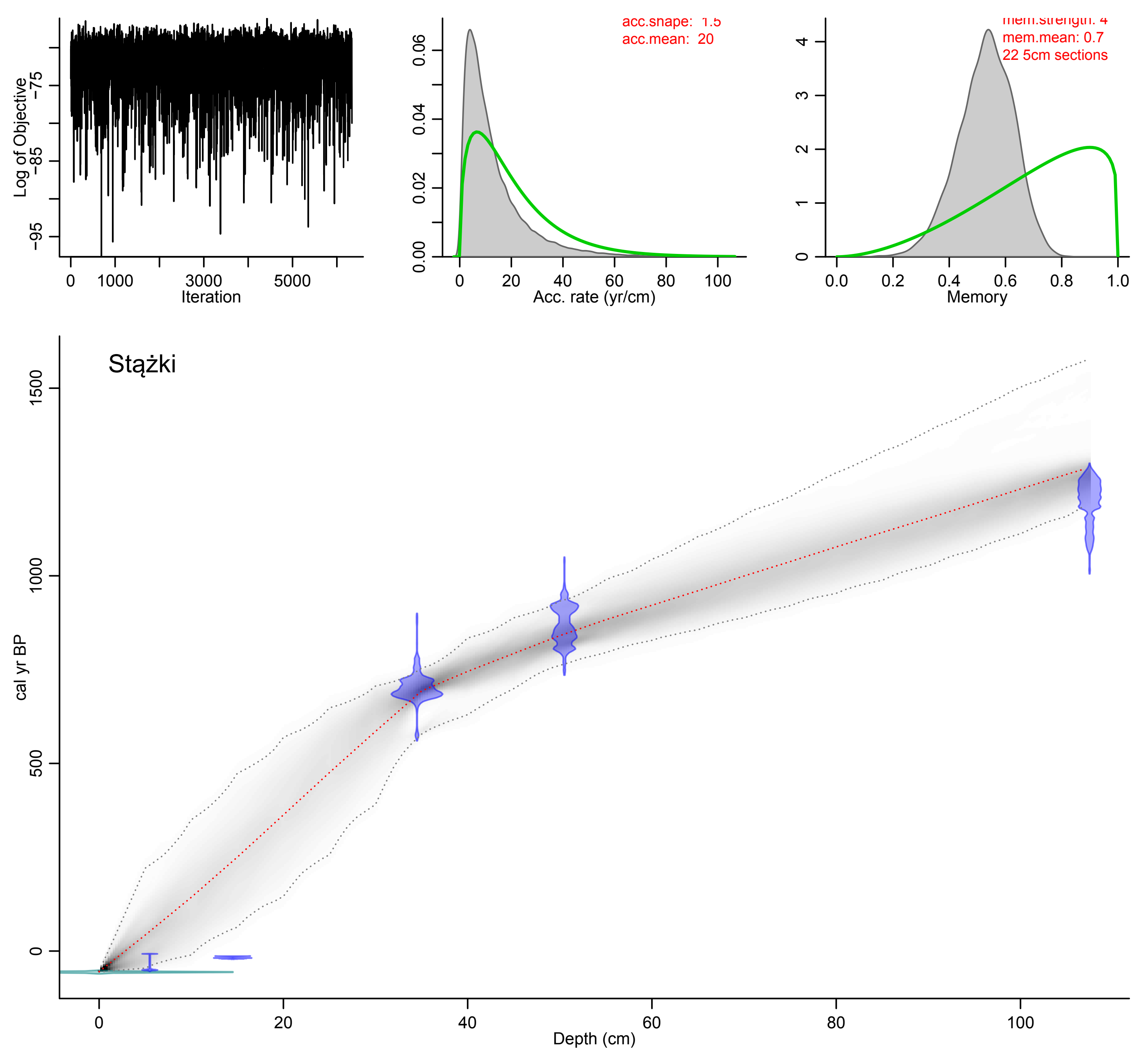

Log of Objective
−95
−85
−75
Iteration
0
1000
3000
5000
acc.shape: 1.5
acc.mean: 20
Acc. rate (yr/cm)
0.00
0.02
0.04
0.06
0
20
40
60
80
100
mem.strength: 4
mem.mean: 0.7
22 5cm sections
Memory
0.0
0.2
0.4
0.6
0.8
1.0
0
1
2
3
4
Stążki
cal yr BP
0
500
1000
1500
Depth (cm)
0
20
40
60
80
100

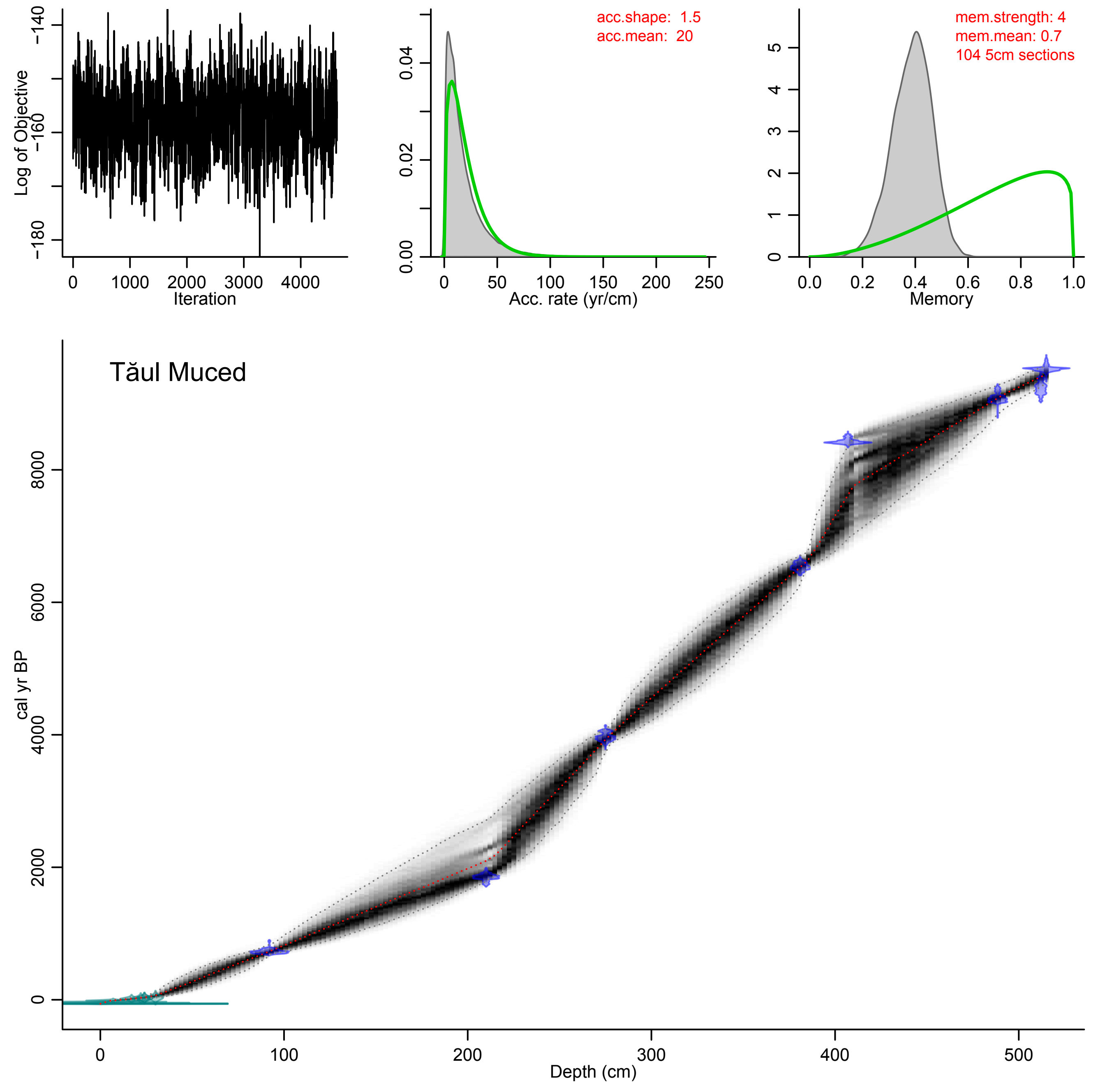

Log of Objective
−140
−160
−180
0
1000
2000
3000
4000
Iteration
acc.shape: 1.5
acc.mean: 20
0.00
0.02
0.04
0
50
100
150
200
250
Acc. rate (yr/cm)
mem.strength: 4
mem.mean: 0.7
104 5cm sections
0
1
2
3
4
5
0.0
0.2
0.4
0.6
0.8
1.0
Memory
Tăul Muced
cal yr BP
0
2000
4000
6000
8000
Depth (cm)
0
100
200
300
400
500

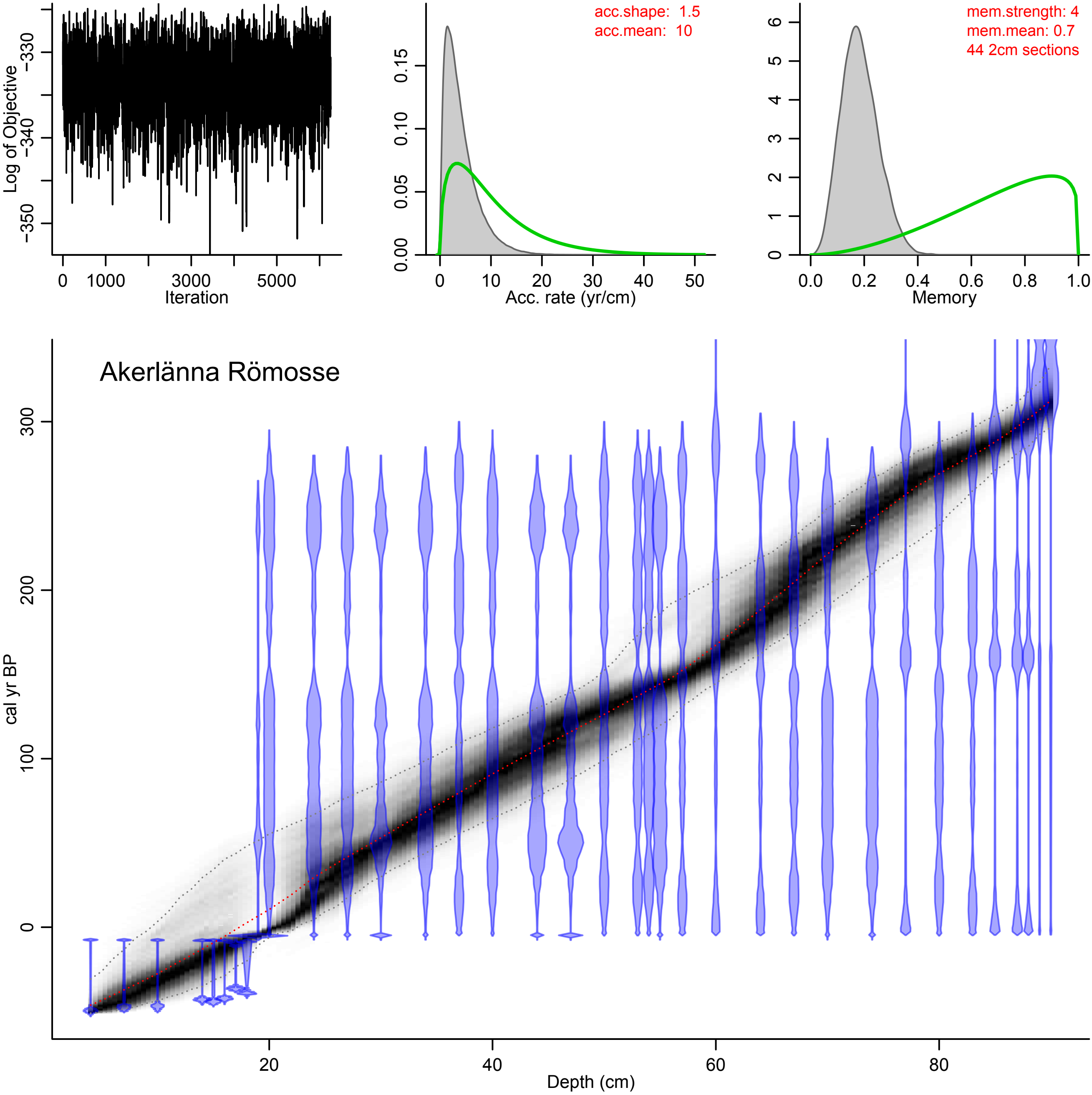

acc.shape: 1.5
acc.mean: 10
mem.strength: 4
mem.mean: 0.7
44 2cm sections
Log of Objective
Iteration
Acc. rate (yr/cm)
Memory
Akerlänna Römosse
cal yr BP
Depth (cm)

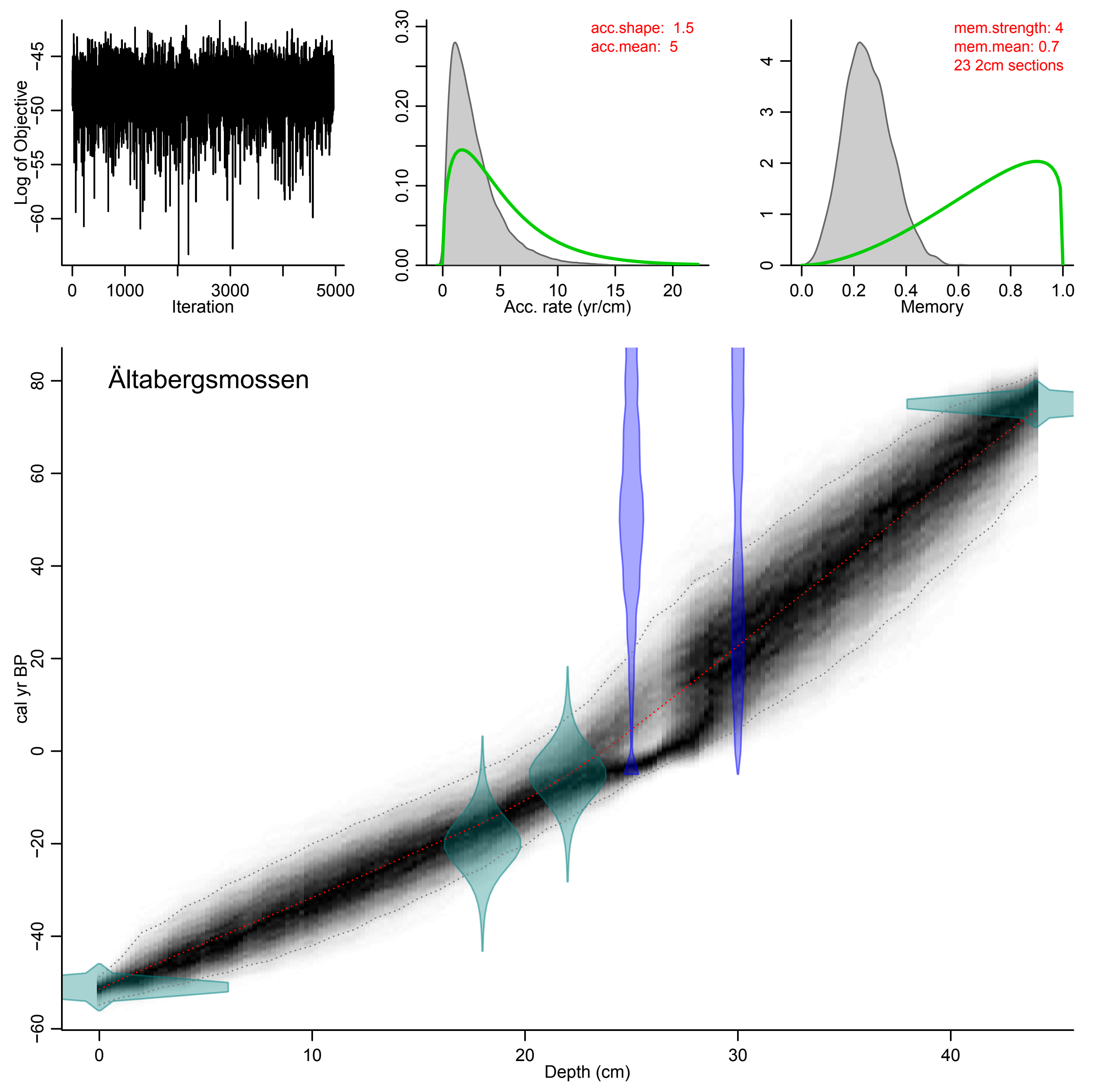

Log of Objective
Iteration
acc.shape: 1.5
acc.mean: 5
Acc. rate (yr/cm)
mem.strength: 4
mem.mean: 0.7
23 2cm sections
Memory
Ältabergsmossen
cal yr BP
Depth (cm)

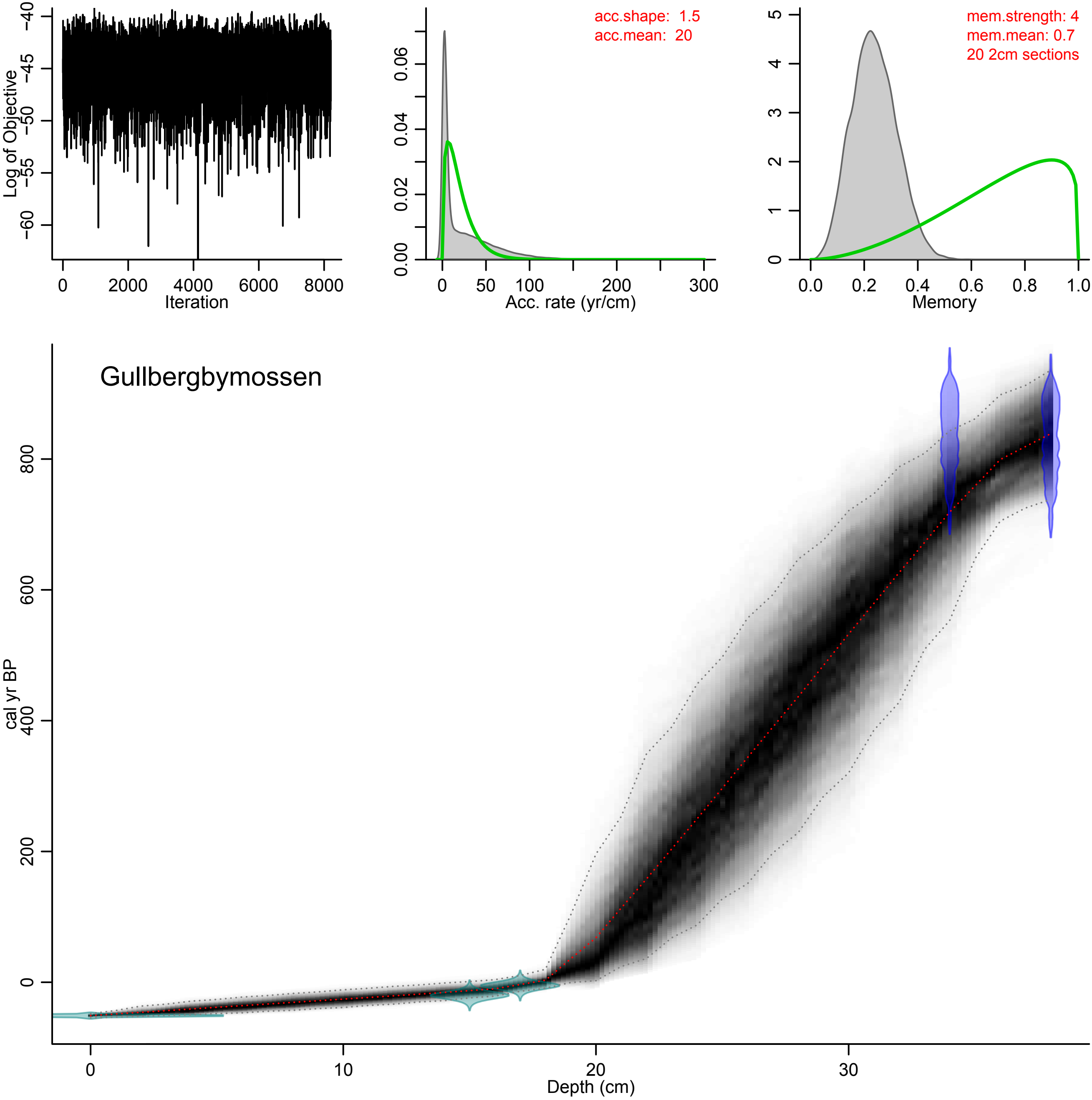

Log of Objective
Iteration
acc.shape: 1.5
acc.mean: 20
Acc. rate (yr/cm)
mem.strength: 4
mem.mean: 0.7
20 2cm sections
Memory
Gullbergbymossen
cal yr BP
Depth (cm)

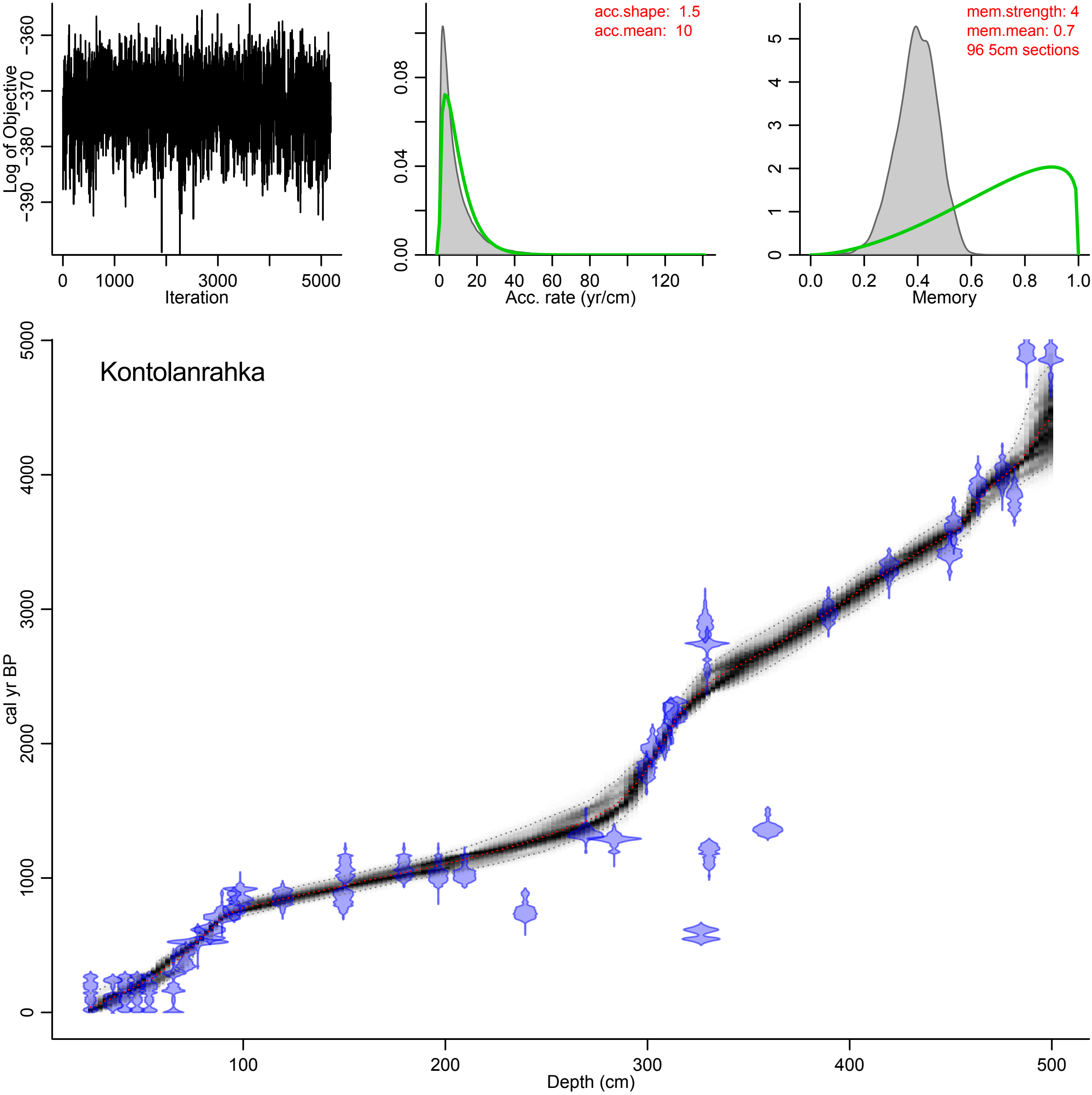

Log of Objective
Iteration
acc.shape: 1.5
acc.mean: 10
Acc. rate (yr/cm)
mem.strength: 4
mem.mean: 0.7
96 5cm sections
Memory
Kontolanrahka
cal yr BP
Depth (cm)

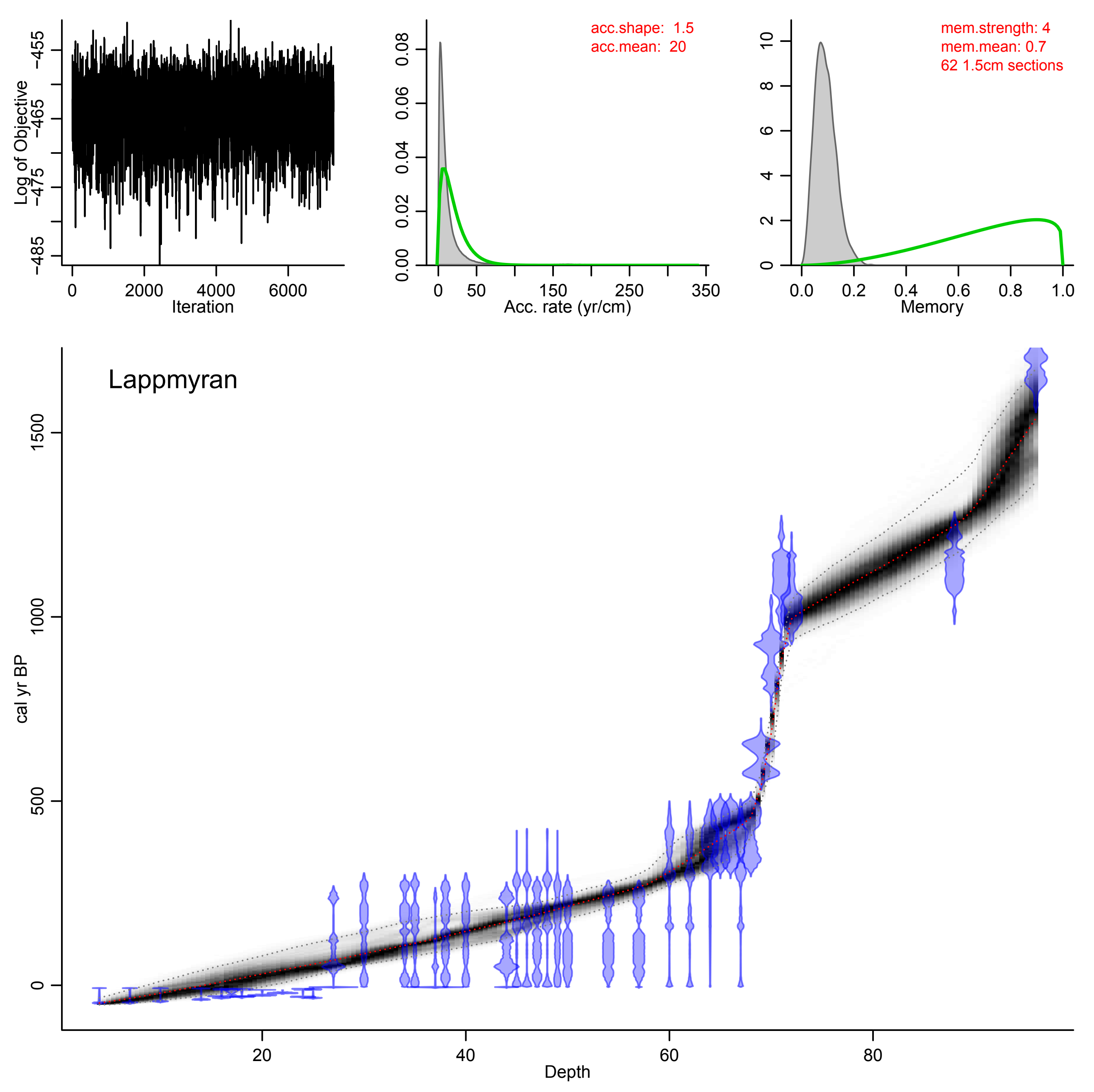

Log of Objective
Iteration
acc.shape: 1.5
acc.mean: 20
Acc. rate (yr/cm)
mem.strength: 4
mem.mean: 0.7
62 1.5cm sections
Memory
Lappmyran
cal yr BP
Depth

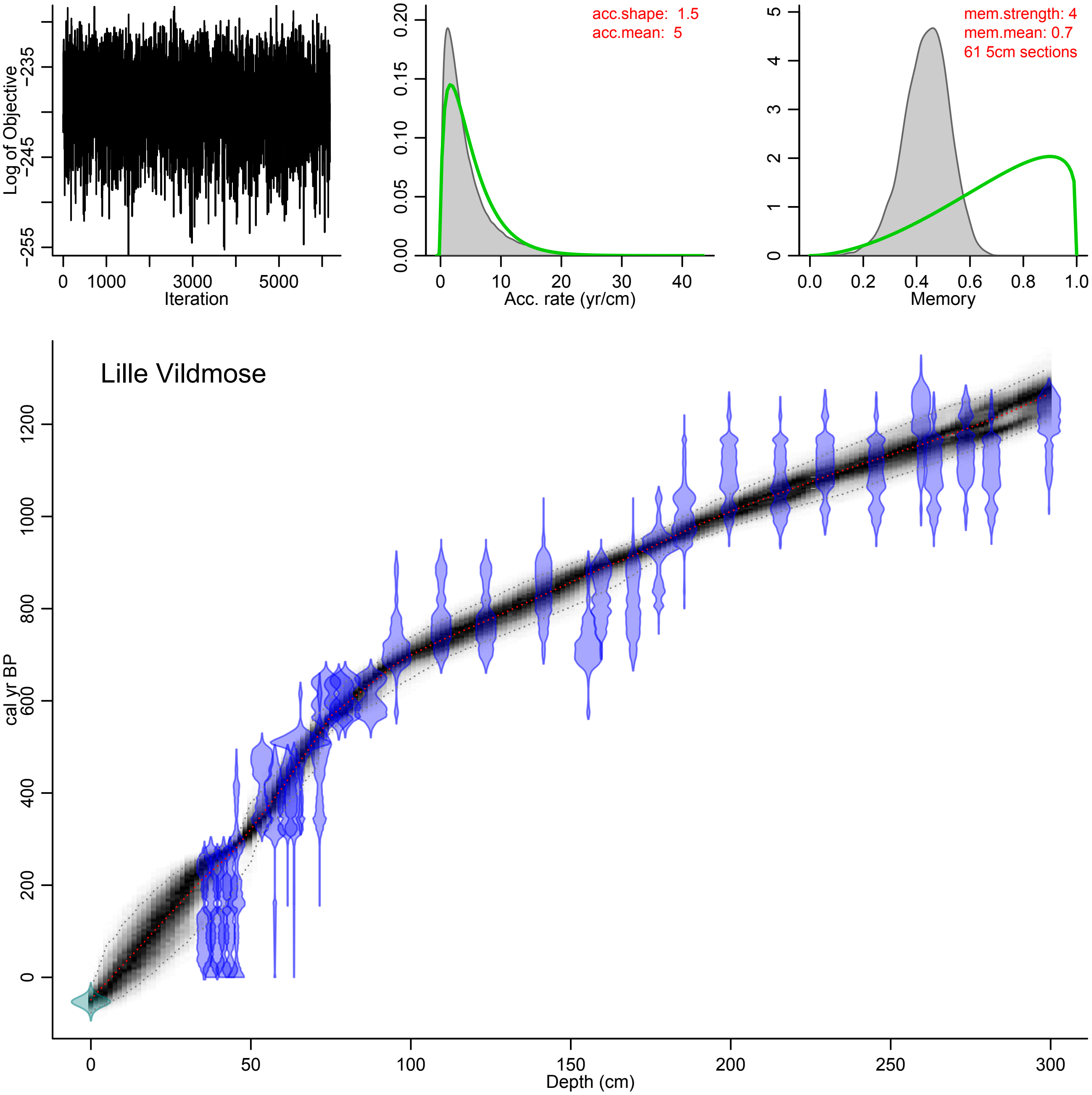

Log of Objective
−235
−245
−255
0
1000
3000
5000
Iteration
0.00
0.05
0.10
0.15
0.20
acc.shape: 1.5
acc.mean: 5
0
10
20
30
40
Acc. rate (yr/cm)
0
1
2
3
4
5
mem.strength: 4
mem.mean: 0.7
61 5cm sections
0.0
0.2
0.4
0.6
0.8
1.0
Memory
Lille Vildmose
cal yr BP
0
200
400
600
800
1000
1200
0
50
100
150
200
250
300
Depth (cm)

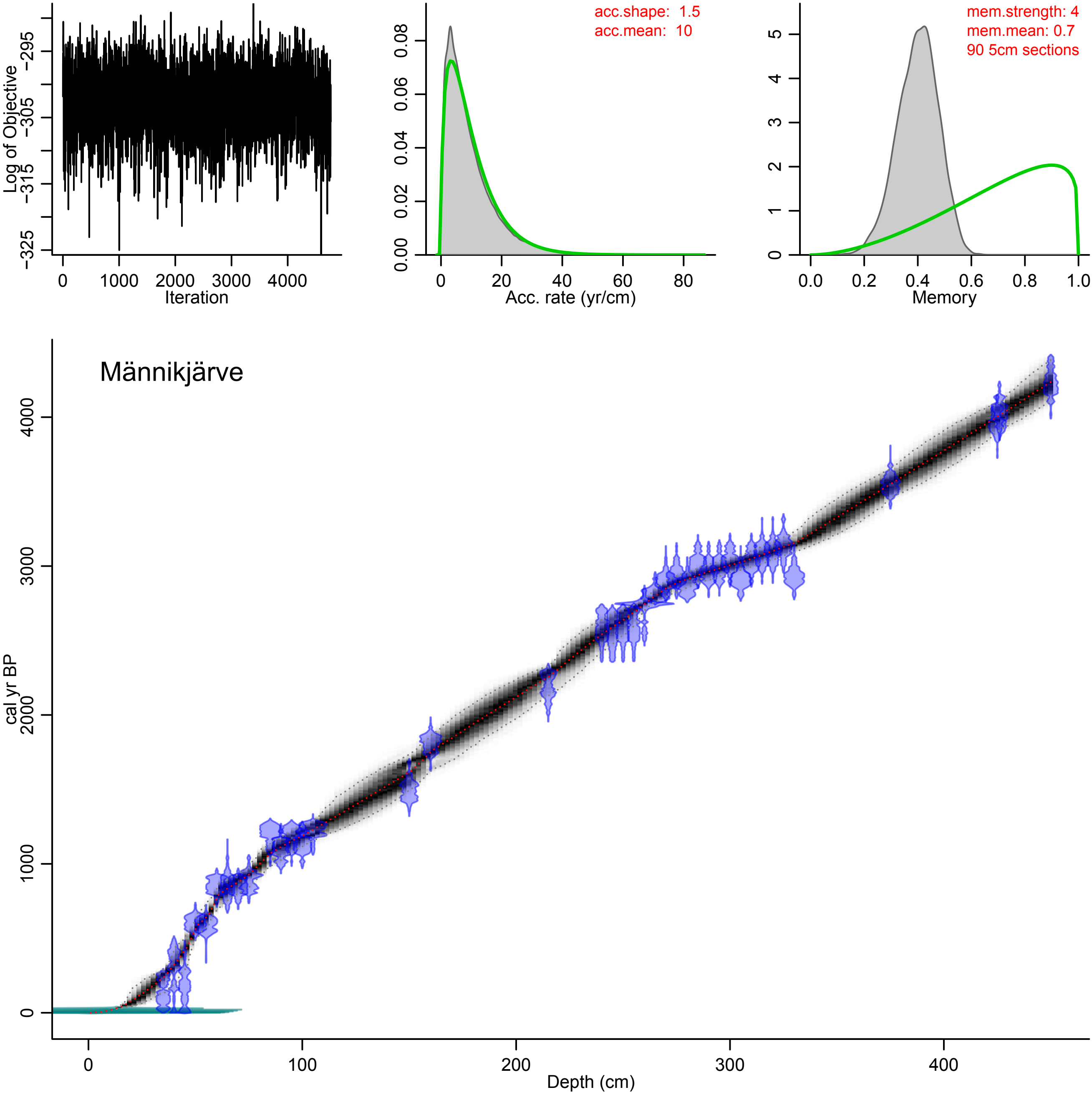
Log of Objective
Iteration
acc.shape: 1.5
acc.mean: 10
Acc. rate (yr/cm)
mem.strength: 4
mem.mean: 0.7
90 5cm sections
Memory
Männikjärve
cal yr BP
Depth (cm)

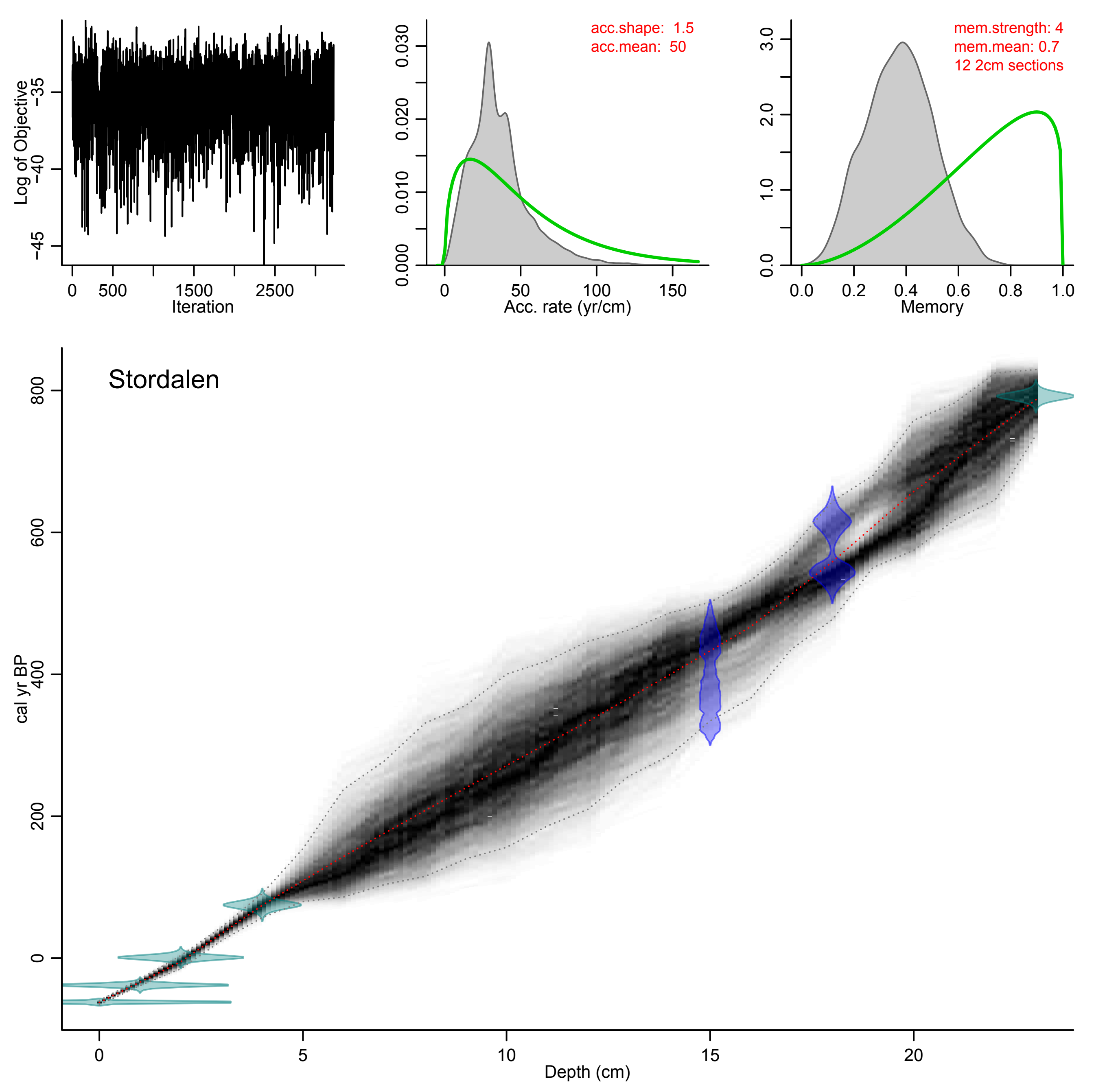

Log of Objective
Iteration
acc.shape: 1.5
acc.mean: 50
Acc. rate (yr/cm)
mem.strength: 4
mem.mean: 0.7
12 2cm sections
Memory
Stordalen
cal yr BP
Depth (cm)
