# Supplementary figures and images for: Climate and water-table levels regulate peat accumulation rates across Europe
Source: PLoS One. 2025 Jul 23;20(7):e0327422. doi: 10.1371/journal.pone.0327422 (PMC12286369; doi:10.1371/journal.pone.0327422)

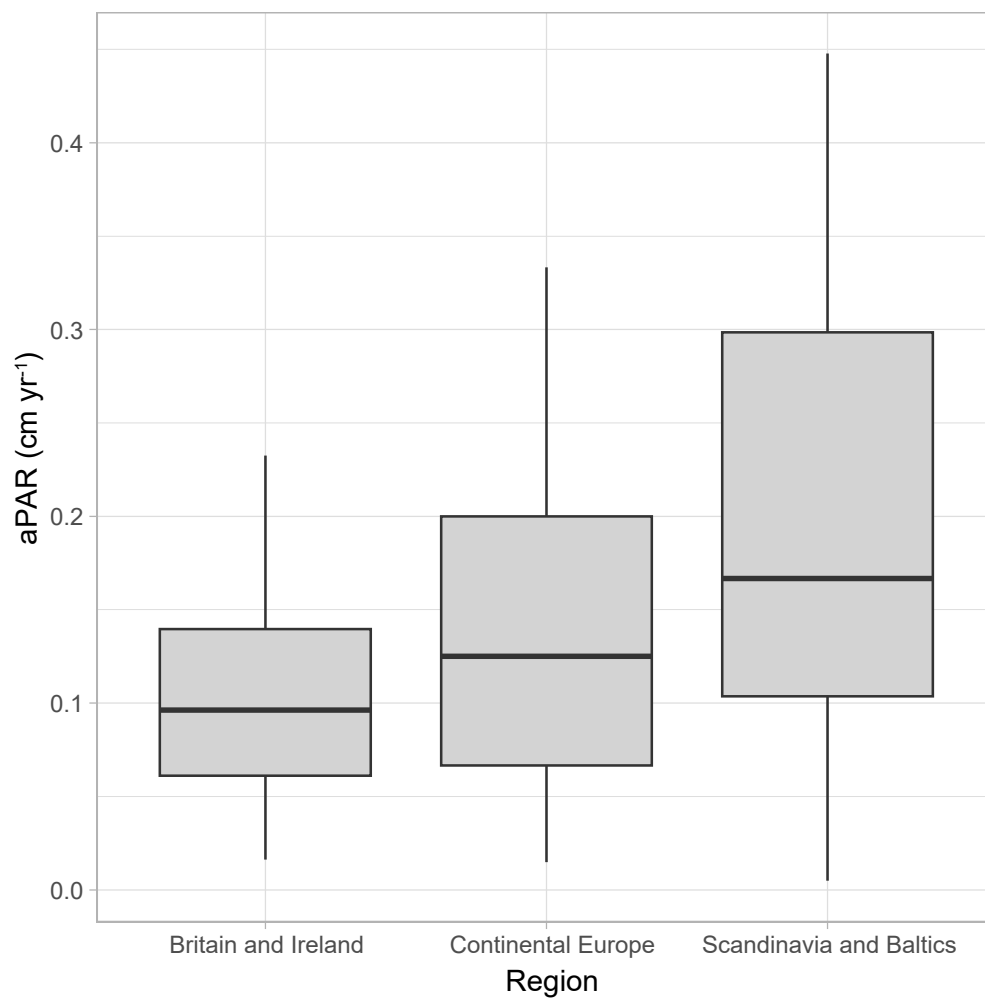

Supplement: S1 File — Supporting Information 1 (Figure): Bayesian age-depth models. Supporting Information 2 (Table): Spearman’s rank correlation information (Rs (bottom left) and p-values (top right) are shown). Supporting Information 3 (Figure): Boxplot showing aPAR for each region (Britain and Ireland, Continental Europe; Scandinavia and Baltics).Supporting Information 4 (Figure): Boxplot showing aPAR across different climate phases including the Little Ice Age (LIA: 1500–1850 CE), Medieval Warm Period (MWP: 950–1250 CE) and Roman Warm Period (RWP: 1–400 CE). All data points are shows as well as the non-Little Ice Age data points (nLIA). Supporting Information 5 (Figure): Theil–Sen robust regression scatterplot of aPAR versus modern climatic data (NOAA–CIRES–DOE 20th Century Reanalysis Version 3). Supporting Information 6 (Figure): Theil–Sen robust regression scatterplot of aPAR versus palaeo-climatic data (CHELSA-TraCE21k). Supporting Information 7 (Table): Site-based data for aPAR, WTD, contemporary climate and palaeo-climate. Supporting Information 8 (Table): Theil–Sen robust regression information for aPAR regressions with WTD, contemporary climate, and palaeo-climate. Supporting Information 9 (Dataset). All data. (ZIP) [file pone.0327422.s001.zip › SI_3_Regions.pdf]

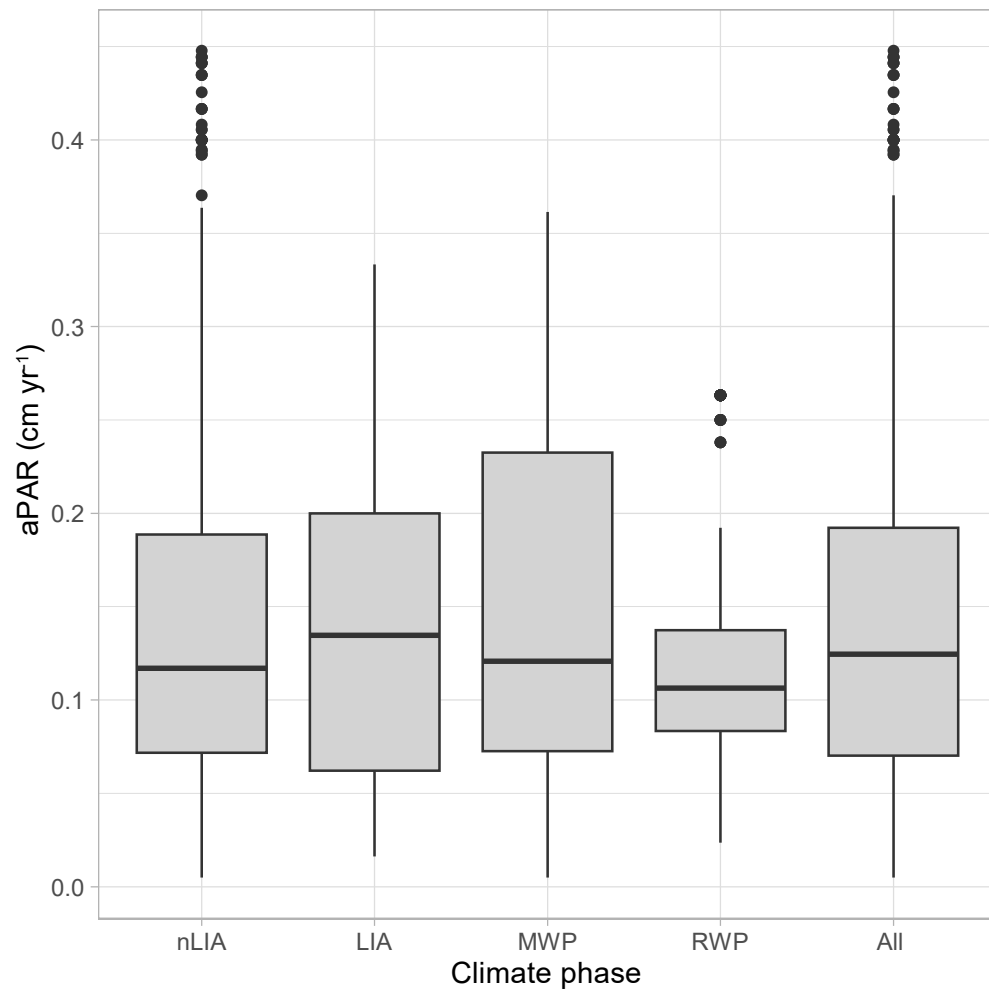

Supplement: S1 File — Supporting Information 1 (Figure): Bayesian age-depth models. Supporting Information 2 (Table): Spearman’s rank correlation information (Rs (bottom left) and p-values (top right) are shown). Supporting Information 3 (Figure): Boxplot showing aPAR for each region (Britain and Ireland, Continental Europe; Scandinavia and Baltics).Supporting Information 4 (Figure): Boxplot showing aPAR across different climate phases including the Little Ice Age (LIA: 1500–1850 CE), Medieval Warm Period (MWP: 950–1250 CE) and Roman Warm Period (RWP: 1–400 CE). All data points are shows as well as the non-Little Ice Age data points (nLIA). Supporting Information 5 (Figure): Theil–Sen robust regression scatterplot of aPAR versus modern climatic data (NOAA–CIRES–DOE 20th Century Reanalysis Version 3). Supporting Information 6 (Figure): Theil–Sen robust regression scatterplot of aPAR versus palaeo-climatic data (CHELSA-TraCE21k). Supporting Information 7 (Table): Site-based data for aPAR, WTD, contemporary climate and palaeo-climate. Supporting Information 8 (Table): Theil–Sen robust regression information for aPAR regressions with WTD, contemporary climate, and palaeo-climate. Supporting Information 9 (Dataset). All data. (ZIP) [file pone.0327422.s001.zip › SI_4_Climate.pdf]

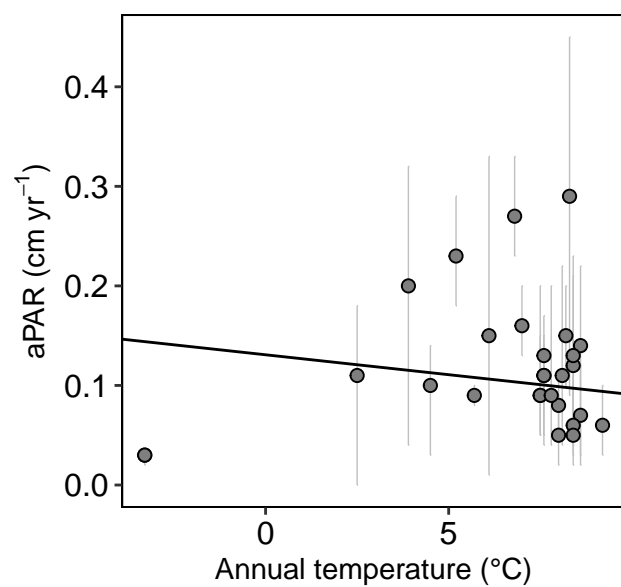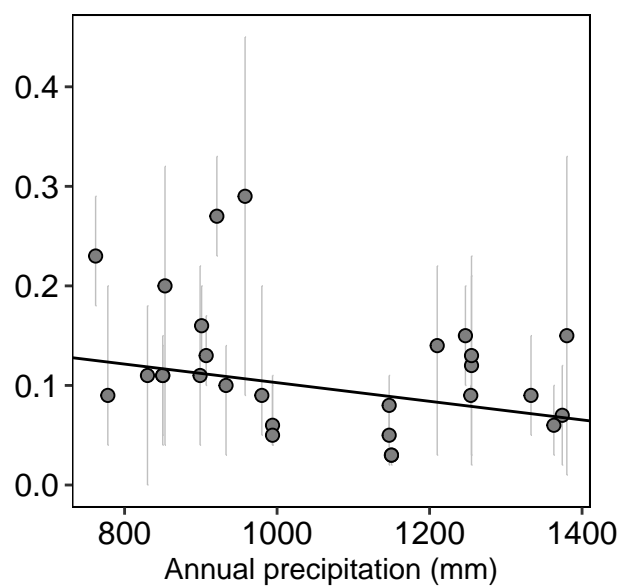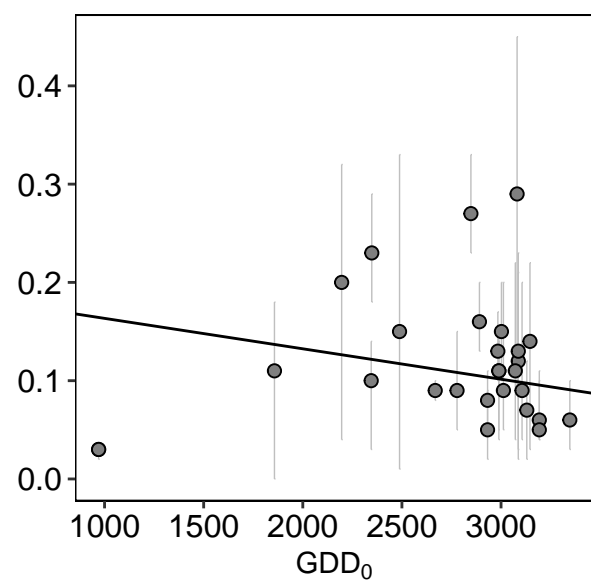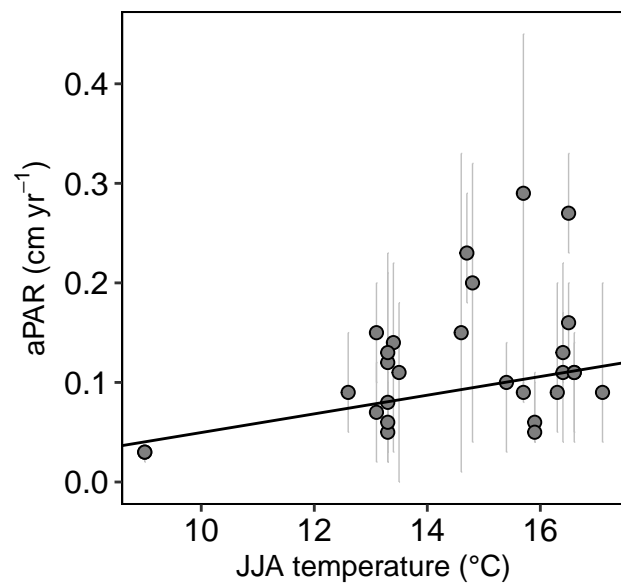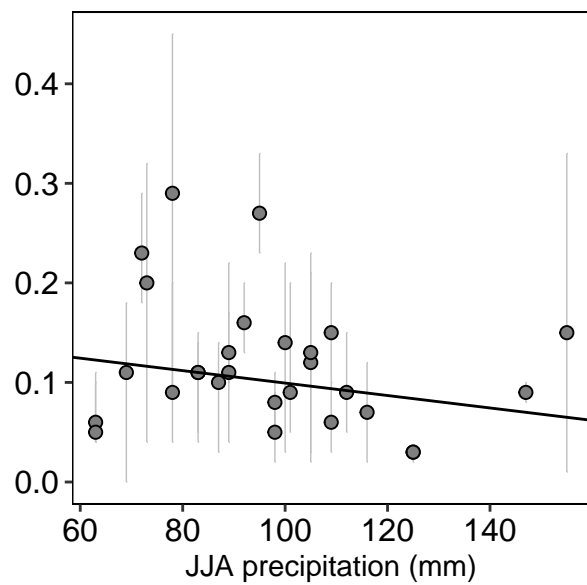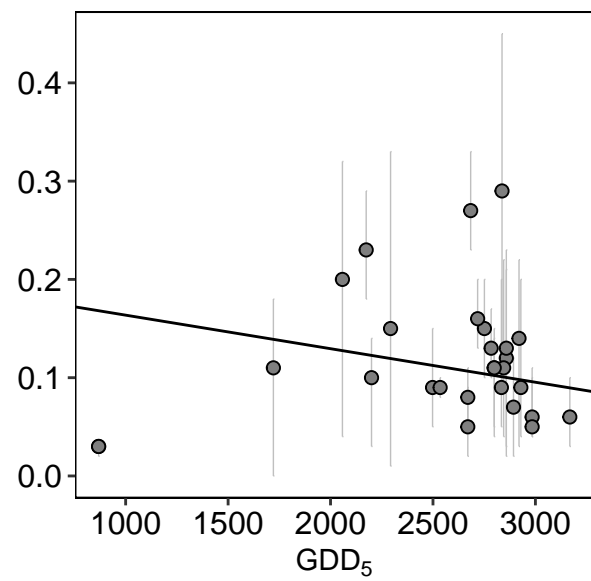

Supplement: S1 File — Supporting Information 1 (Figure): Bayesian age-depth models. Supporting Information 2 (Table): Spearman’s rank correlation information (Rs (bottom left) and p-values (top right) are shown). Supporting Information 3 (Figure): Boxplot showing aPAR for each region (Britain and Ireland, Continental Europe; Scandinavia and Baltics).Supporting Information 4 (Figure): Boxplot showing aPAR across different climate phases including the Little Ice Age (LIA: 1500–1850 CE), Medieval Warm Period (MWP: 950–1250 CE) and Roman Warm Period (RWP: 1–400 CE). All data points are shows as well as the non-Little Ice Age data points (nLIA). Supporting Information 5 (Figure): Theil–Sen robust regression scatterplot of aPAR versus modern climatic data (NOAA–CIRES–DOE 20th Century Reanalysis Version 3). Supporting Information 6 (Figure): Theil–Sen robust regression scatterplot of aPAR versus palaeo-climatic data (CHELSA-TraCE21k). Supporting Information 7 (Table): Site-based data for aPAR, WTD, contemporary climate and palaeo-climate. Supporting Information 8 (Table): Theil–Sen robust regression information for aPAR regressions with WTD, contemporary climate, and palaeo-climate. Supporting Information 9 (Dataset). All data. (ZIP) [file pone.0327422.s001.zip › SI_5thiel_sen_scatterplot_Modern_climate_APR_25.pdf]

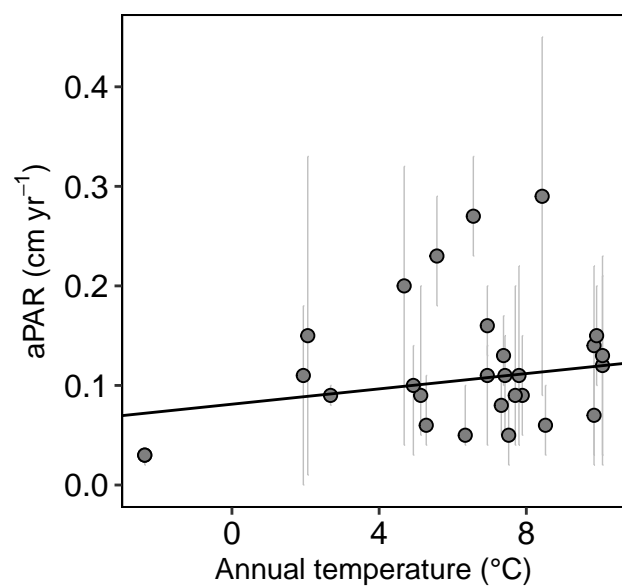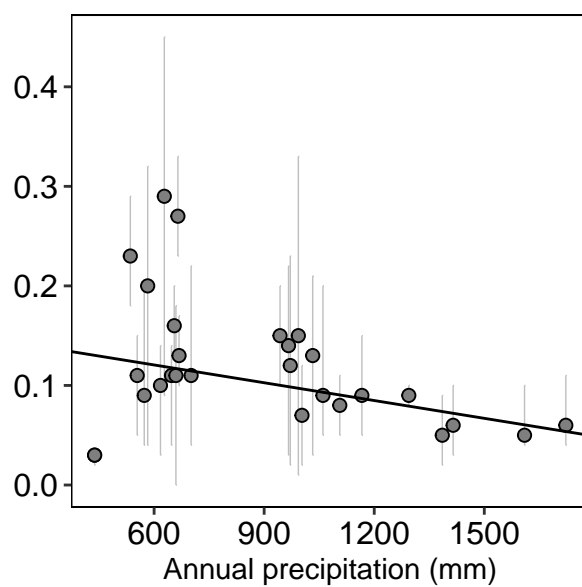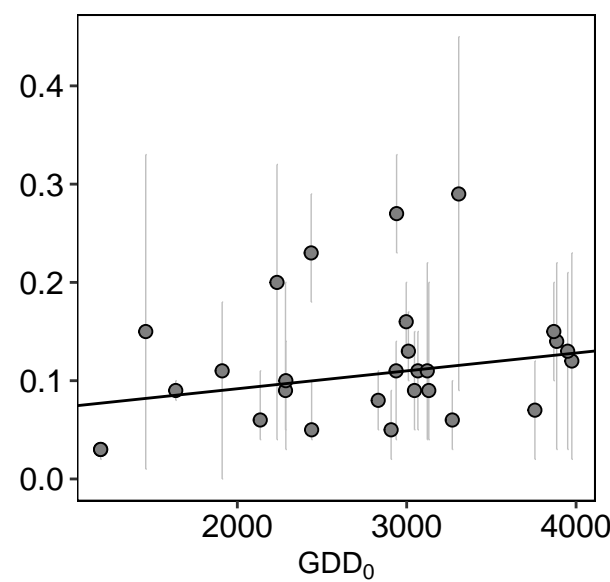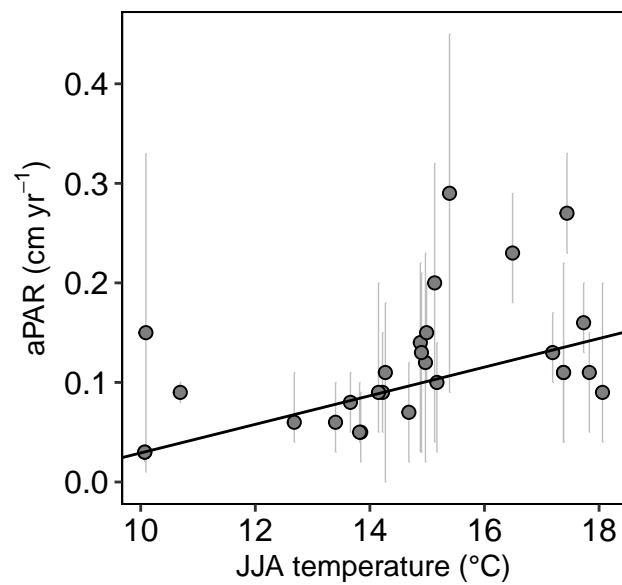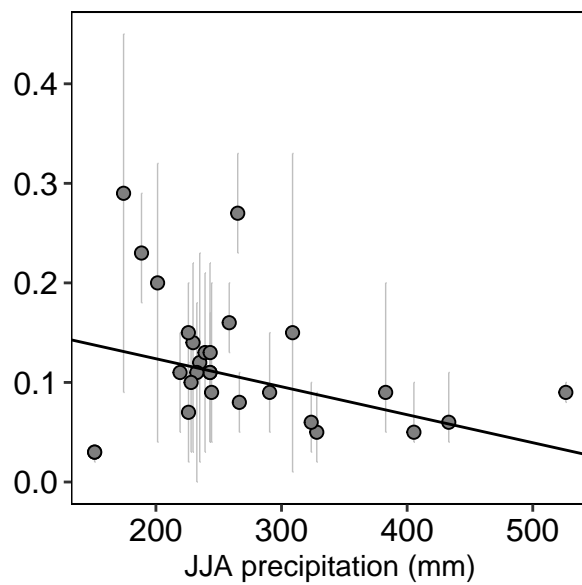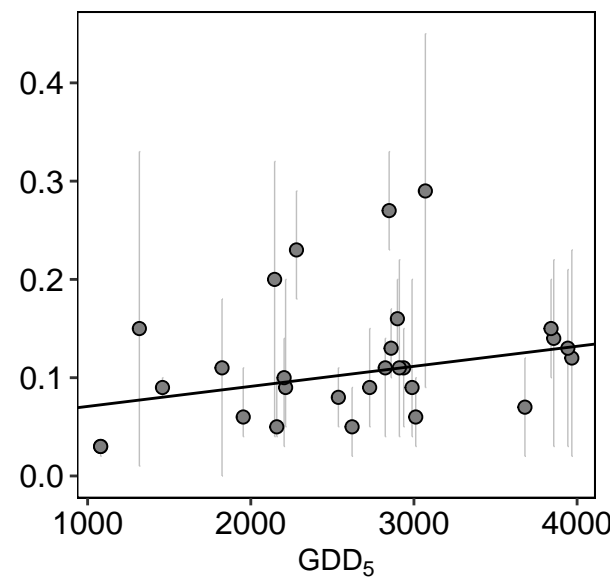

Supplement: S1 File — Supporting Information 1 (Figure): Bayesian age-depth models. Supporting Information 2 (Table): Spearman’s rank correlation information (Rs (bottom left) and p-values (top right) are shown). Supporting Information 3 (Figure): Boxplot showing aPAR for each region (Britain and Ireland, Continental Europe; Scandinavia and Baltics).Supporting Information 4 (Figure): Boxplot showing aPAR across different climate phases including the Little Ice Age (LIA: 1500–1850 CE), Medieval Warm Period (MWP: 950–1250 CE) and Roman Warm Period (RWP: 1–400 CE). All data points are shows as well as the non-Little Ice Age data points (nLIA). Supporting Information 5 (Figure): Theil–Sen robust regression scatterplot of aPAR versus modern climatic data (NOAA–CIRES–DOE 20th Century Reanalysis Version 3). Supporting Information 6 (Figure): Theil–Sen robust regression scatterplot of aPAR versus palaeo-climatic data (CHELSA-TraCE21k). Supporting Information 7 (Table): Site-based data for aPAR, WTD, contemporary climate and palaeo-climate. Supporting Information 8 (Table): Theil–Sen robust regression information for aPAR regressions with WTD, contemporary climate, and palaeo-climate. Supporting Information 9 (Dataset). All data. (ZIP) [file pone.0327422.s001.zip › SI_6thiel_sen_scatterplot_Palaeo_climate_APR_25.pdf]
