# Supplementary material for: Climate and water-table levels regulate peat accumulation rates across Europe
Source: PLoS One. 2025 Jul 23;20(7):e0327422. doi: 10.1371/journal.pone.0327422 (PMC12286369; doi:10.1371/journal.pone.0327422)
Supplement: Attachment — Submitted filename: RESPONSE TO REVIEWERS.docx [file pone.0327422.s003.docx]

**RESPONSE TO REVIEWERS:**

Reviewer #1: This is an interesting manuscript that provides significant insights into peat accumulation rates across 28 well-dates European mires that are at least 2000 years old. The findings are particularly important for peat restoration efforts, particularly in the face of current and future climate change, suggesting that peat accumulation rates are controlled by complex interactions between climate, vegetation type and water depth, as well as finding a degree of geographic variation (potentially linked to these factors) across the study sites. A key finding is that 10 cm water table depths is crucial for peat restoration efforts and that further research is needed to underpin restoration efforts, particularly in terms of complexity of peat accumulation rates and how this can be applied to restoration efforts. Based on this I strongly support publication of this manuscript, as these findings will be of broad interest, not only to European researchers, but also other researchers across the globe as this provides an approach that will be directly relevant to other peatland systems, particularly in terms of underpinning restoration efforts of these vitally important ecosystems. There are a few minor issues (mainly related to referencing) that need to be addressed (listed below), and once these have been rectified the manuscript is ready for publication.

We would like to thank the reviewer for their positive review of our work.

Line 159 to 160 - Dommain et al., 2010 listed with a publication year of 2016 in references (lines 456 to 461) - please verify the correct publication year.

Fixed.

Line 230 - Turetsky et al., 2010 listed with a publication year of 2015 in references (lines 584 to 585) - please verify the correct publication year.

Fixed.

Line 237 - Dorrepaal et al., 2003 listed with a publication year of 2004 in references (lines 462 to 464) - please verify the correct publication year.

Fixed.

##########################

Reviewer #2: Review of Swindles et al. PONE-D-24-48416 “Do climatic or hydrological factors influence peat accumulation rates across Europe?” Plos One.

Manuscript status

This manuscript is the first submission.

Comments

The manuscript aimed “to determine the climatic and hydrological controls on the apparent rates of peat accumulation (aPAR)” using “palaeoenvironmental data from 28 well-dated, intact European peat bogs”. The aPAR was analysed over the last 2000 years. The main finding is that the aPAR ranged between 0.005 and 0.448 cm yr-1. Although weak, there was a significant relationship between aPAR and water table depth (WTD) with an optimal aPAR at a WTD of -10 cm, which confirms the common result observed on shorter time series than in this study. The study is impressive in gathering data from 28 sites in Europe, this is solid.

We thank the reviewer for positive comments on our manuscript.

However, there is a fundamental issue in the methods to estimate the relationship between aPAR and climatic variables relative to the relationship between aPAR and WTD:
L341-359. When reading the M&M on the WTD and climate reconstruction, it is understood that climate analysis was reconstructed for the period 1836-2015 while WTD reconstruction was done for the full peatland life durations with the subfossil testate amoebae. The reconstruction periods are different for both climate and WTD. It also means that climate does not have the same reconstruction range than WTD when examining the relationships with aPAR. Moreover, the M&M section shows that the climate reconstruction period 1836-2015 is almost not included in the aPAR period as the aPAR did not consider the peat accumulation since 1850AD (L339).

Is there something not explained in the M&M for the fitting between aPAR and climate? At first reading, it seems that the fitting is impossible as aPAR since 1850 is removed while climate data were available for 1836-2015. So, strictly reading the description indicates that the reconstruction could not be done between aPAR and climate as the time periods do not coincide. It seems impossible because as a large part of the work is on this relationship. Something is really unclear. Was there a climate reconstruction before 1836 to be fitted with the aPAR? Otherwise, what time period was concretely and clearly used in the relationship between aPAR and climate? This comment is major as a large part of the manuscript concerns the relationship between aPAR and climate.

The reviewer has made an excellent comment regarding the chronological mismatch of palaeodata against contemporary climate data. To address this fully, we have re-done part of the analysis using the *CHELSA-TraCE21k* long-term climate record covering the period 100 B.C.E to 1990 C.E. (Karger et al., 2023). This has made the analysis robust and has also shown Summer Temperature as a stronger control on aPAR than we found based on the contemporary climate data alone.

Methodological information added:

Mean monthly and annual palaeo-climate simulation data were taken from the CHELSA-TraCE21k long-term climatology covering the period 100 B.C.E to 1990 C.E. (Karger et al., 2023). The data are available at 100-year timesteps at high spatial resolution. We downloaded these data from: https://chelsa-climate.org/chelsa-trace21k/. Mean annual temperature (°C) and total annual precipitation (mm yr-1) data were taken directly from the variables bio1 and bio12. Mean monthly temperature was calculated from the mean of tasmin and tasmax. Mean monthly temperature and precipitation totals were then aggregated for northern hemisphere summer (June, July, August) for each 100-yr timestep. For GDD0 and GDD5, we multiplied mean monthly temperatures by the days of each month and summed these values relative to thresholds of 0 °C and 5 °C respectively.

There is another minor comment:
In L121-124, the information on the WTD threshold vs peat accumulation rates is well synthesised in the abstract but it does not appear as concise in the results in L194-207 where only results for the WTD of 5-11 cm is shown. The < 0 cm and > 25 cm thresholds are also not discussed or presented in the discussion.

Therefore, either change the text here in the abstract to reflect the results or add details in the results and discussion to indicate the relationship or threshold for the < 0 cm and > 25 cm effects. The minor issue here is why we read on thresholds of < 0 cm and > 25 cm in the abstract while it does not appear at all in the manuscript.

Fixed. This has now been addressed with text added to the results and discussion sections.

Apart from these two comments, the manuscript was well written and structured.

Many thanks for these kind comments on our manuscript.

Note to the editor for question 3: it is not possible to answer as we do not see where data would be available in the web portal. I find that enough data are available through the supplementary information, no need for more.

Many thanks for clarification. All data will be added as a supplementary file to the paper.
